# Supplementary material for: Multi-level and lineage-specific interactomes of the Hox transcription factor Ubx contribute to its functional specificity
Source: Nat Commun. 2020 Mar 13;11:1388. doi: 10.1038/s41467-020-15223-x (PMC7069958; doi:10.1038/s41467-020-15223-x)
Supplement: Supplementary file 1 — Supplementary Information [file 41467_2020_15223_MOESM1_ESM.pdf]

Supplementary Information

Multi-level and Lineage-Specific Interactome of the Hox Transcription Factor Ubx Contributes to its Functional Specificity

Carnesecchi et al.

SUPPLEMENTARY NOTE

Supplementary note 1: Validation of the BioID in Drosophila cell system. In order to verify the suitability of BioID for identifying Ubx interaction partners, we expressed the different mB* fusion constructs in biotin supplemented *Drosophila* S2R+ cells. Subsequent streptavidin purification and Western blot analysis revealed that all mB* fusion proteins were biotinylated by BirA* (Supplementary Figs 1b, 1c). Furthermore, mB*Ubx expression was associated with a strong global biotinylation after 30 min of biotin supplementation (Supplementary Fig. 1d). This finding was of particular relevance, as fast biotinylation is required to detect close-proximity interactions *in vivo*. Importantly, we detected two known Ubx interactors, exogenously expressed Extradenticle (Exd)[1] and endogenously expressed Mediator complex subunit 19 (Med19)[2], in the streptavidin purified fraction (Supplementary Fig. 1b).

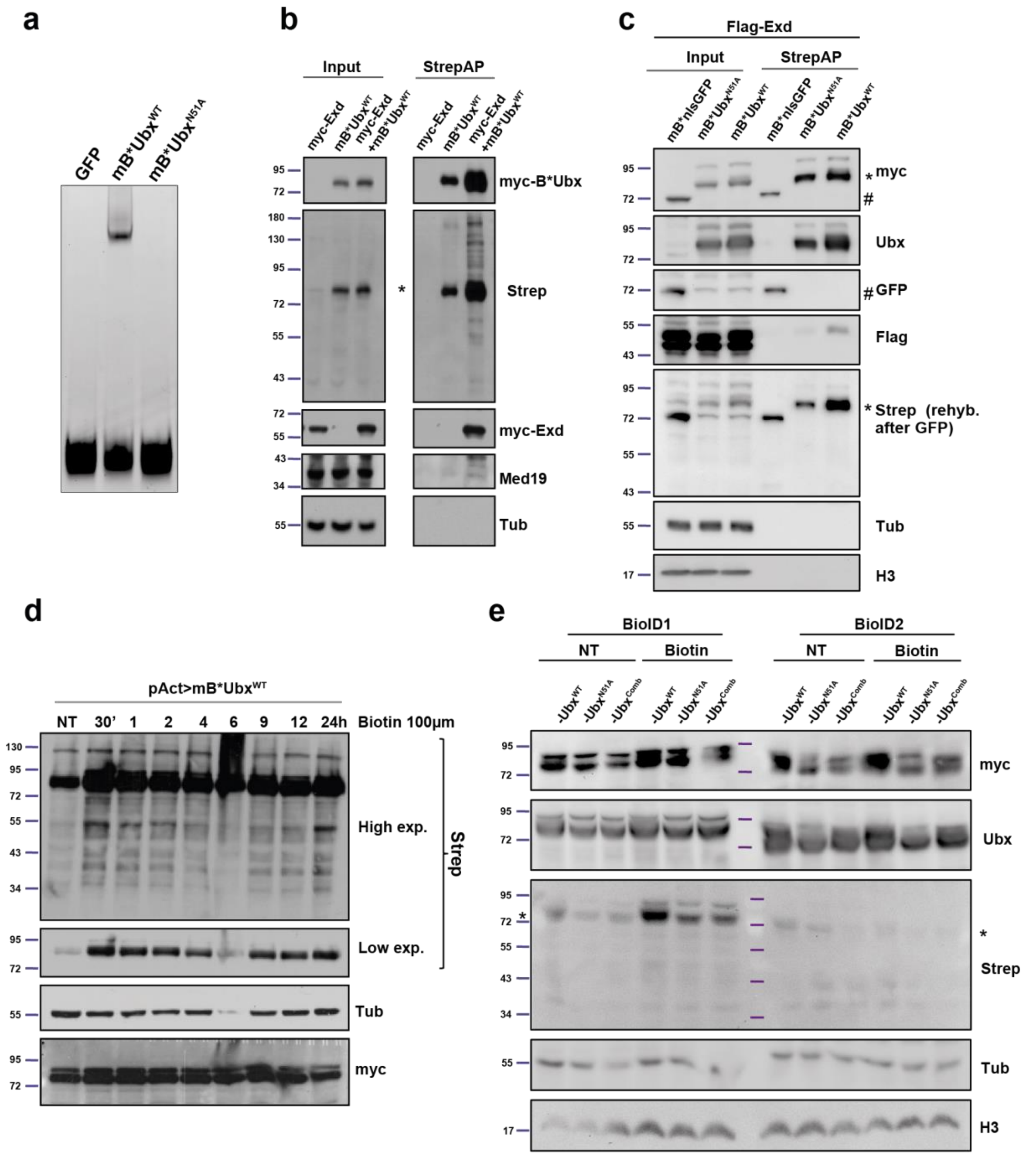

Supplementary Figure 1: Establishment of BioID in *Drosophila* S2R+ cells.

(a) EMSA with purified proteins (GFP, mB*Ubx^{WT} and mB*Ubx^{N51A}) on Ubx consensus sequence, showing that the single amino acid exchange in the Ubx homeodomain (Ubx^{N51A}) circumvents DNA binding of Ubx. **(b, c)** Streptavidin affinity purification (BioID) performed on indicated cell extracts. The input (lane 1-3) and the streptavidin purified fraction (StrepAP) are shown. Ubx, GFP, myc, Med19, Streptavidin (Strep) and Tubulin (Tub) antibodies were used for detection. The asterisks indicate biotinylated Ubx. myc-Exd and Med19 (a) were specifically pulled down in StrepAP fraction upon co-expression with mB*Ubx^{WT} (lane 6 compared lane 4). Flag-Exd (b) was specifically pulled down in StrepAP fraction with mB*Ubx^{WT} and to a lesser extent with mB*Ubx^{N51A} (lane 6 and 5 compared to lane 4). The

ratio of expression of N51A:WT Ubx (input) is ‘0.8:1’, indicating a rather similar level of expression of the constructs. In contrast, affinity purification of Exd (normalized to expression in the Input) in the N51A and WT fraction is ‘0.3:1’, showing a stronger biotinylation of Exd by Ubx^{WT} than Ubx^{N51A}. This indicates that close-proximity events happen more often between Exd-Ubx^{WT} than between Exd-Ubx^{N51A}. **(d)** Immunoblots of global biotinylation of cells transfected with pActin-Gal4 and UAS-mB*Ubx^{WT}, which were treated with Biotin for the indicated times. Low and high exposures of Streptavidin (Strep) are shown as well as myc (for mB*Ubx) and Tubulin (Tub) as internal controls. **(e)** Immunoblots showing global biotinylation of cells transfected with pActin-Gal4, UAS-mB* (BioID1) or BioID2 (from *A. aeolicus*) fused with Ubx^{WT}, Ubx^{N51A}, Ubx^{Comb} (Ubx mutant version with four point mutations in the HD, which did not circumvent DNA binding). Cells are treated (or not) with 100µM of Biotin. Ubx, myc, Histone 3 (H3), Tubulin (Tub) and Streptavidin (Strep) antibodies were used for detection. Asterisks indicate Ubx. Regular BirA* (BioID1) induced stronger biotinylation than BirA* from *A. aeolicus* (BioID2) (compared lanes 4-6 to lanes 10-12). Protein size is indicated relative to ladder position.
Source files are provided in Source-Data File.

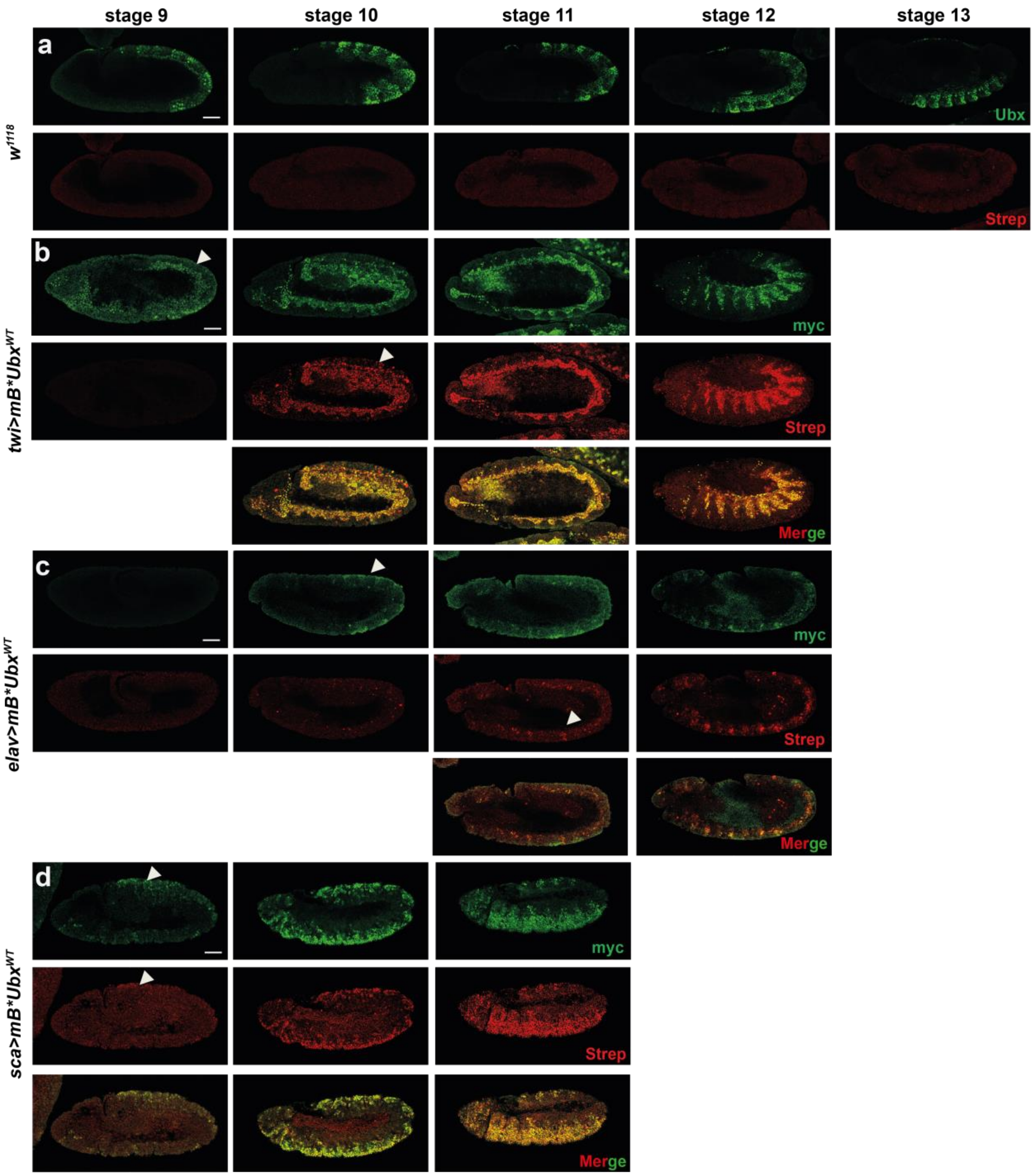

Supplementary Figure 2: Tissue-specific expression and biotinylation using BioID.

(a) Immunostaining of stages 9, 10, 11, 12 and 13 w^{1118} embryos with Ubx (green) and Streptavidin (red). Expression of Ubx can be detected from stage 9 on, while no biotinylation is observed. **(b)** Immunostaining of stage 9, 10, 11 and 12 embryos expressing *twi-GAL4>UAS-mB*Ubx*^{WT}. Transgene expression is shown by myc staining (green), biotinylated proteins by Streptavidin staining (red), the merge highlights the overlap. Expression of mB*Ubx is detected from stage 9 on, while biotinylation in only detected significantly at stage 10 (see white arrowhead). **(c)** Immunostaining of stage 9, 10, 11 and 12 embryos expressing *elav-GAL4>UAS-mB*Ubx*^{WT}. Expression of mB*Ubx is detected from stage 10 on, while

biotinylation is only detected significantly at stage 11 (see white arrowhead). **(d)** Immunostaining of stage 8, 9, 10 and 11 embryos expressing _sca-GAL4>UAS-mB*Ubx_^{WT}. Expression of mB*Ubx as well as biotinylation is detected from stage 9 on (see white arrowhead). Images are representative of all embryos analysed per genotype over 3 sets of embryos collection from independent crossings.
Scale bar=50µm.

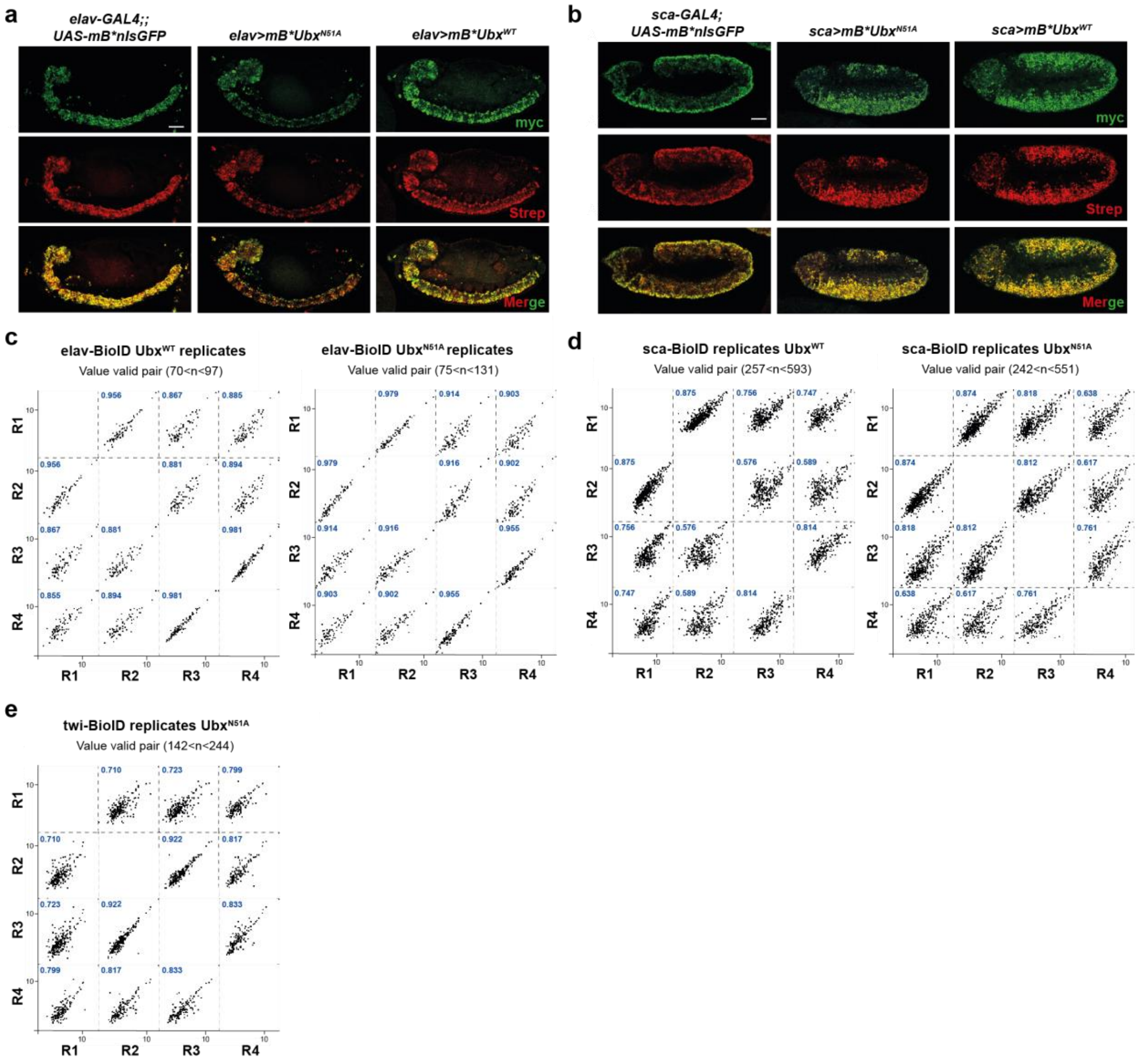

Supplementary Figure 3: Validation of targeted BioID in *Drosophila* embryonic tissues. (a, b) Immunostaining of stage 13 (5-8h AEL) (a) and stage 10 (2.5-5h) (b) embryos, which express the *UAS-mB*nlsGFP* (*left panels*), *UAS-mB*Ubx*^{N51A} (*middle panels*) or *UAS-mB*Ubx*^{WT} (*right panels*) transgenes in the neural system by means of the *elav-GAL4* driver (a) and in the neuroectodermal system by means of the *sca-GAL4* driver (b). Transgene expression is shown by myc staining (green), biotinylated proteins are visualised by Streptavidin staining (red), the merge highlights the specificity of the biotinylation. **(c, d)** Pearson correlation (blue) and valid value of four replicates of elav-BioID (c) and sca-BioID (d) samples of Ubx^{N51A} and Ubx^{WT} are presented. **(e)** Pearson correlation (blue) and valid value of four replicates of twi-BioID samples of Ubx^{N51A} is shown.

Images are representative of all embryos analysed per genotype over 3 sets of embryos collection from independent crossings.

Scale bar=50µm.

a

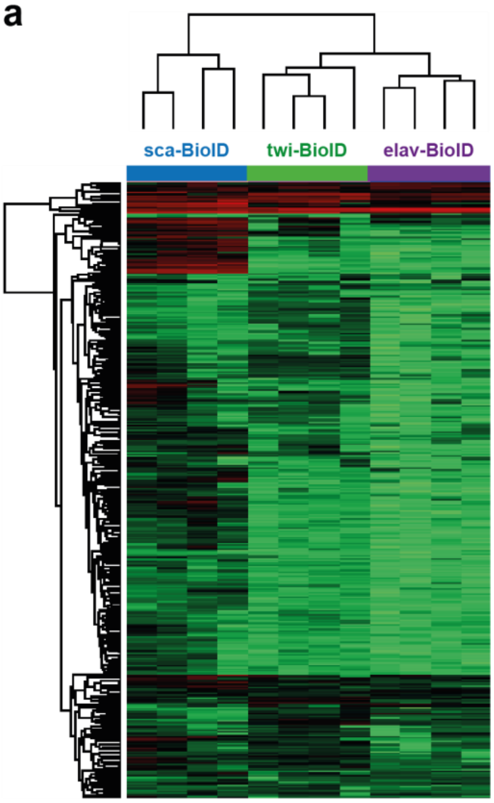

b

	Pearson	elav (0.66)			
	Replicate	1	2	3	4
twi-BioID	1	.682	.648	.609	.606
	2	.633	.715	.653	.652
	3	.655	.743	.667	.66
	4	.679	.697	.639	.628

	Pearson	sca (0.328)			
	Replicate	1	2	3	4
elav-BioID	1	.278	.316	.231	.311
	2	.305	.334	.319	.41
	3	.303	.288	.343	.416
	4	.299	.306	.324	.399

	Pearson	twi (0.245)			
	Replicate	1	2	3	4
sca-BioID	1	.1	.218	-.019	.149
	2	.341	.392	.267	.406
	3	.273	.362	.193	.326
	4	.224	.319	.098	.243

Supplementary Figure 4: Comparison of tissue-specific Ubx interactomes.

(a) Clustering and heatmap based on LFQ value of proteins identified after Perseus filtering in four replicates of the sca-, twi- and elav-BioIDs from Ubx^{WT} samples. **(b)** Table of Pearson correlations and averages between the four BioID replicates of the different tissues for Ubx^{WT} samples. See also Supplementary Data 1-3.

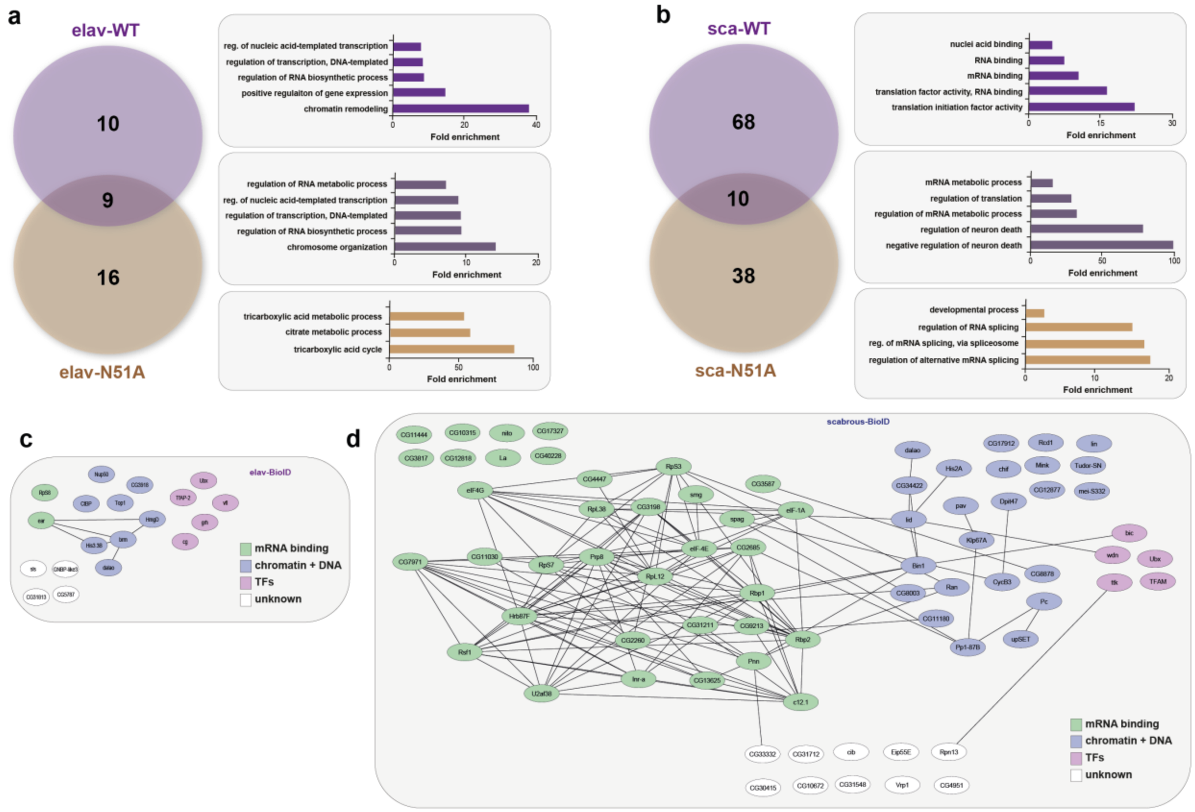

Supplementary Figure 5: Analysis of neural and neuroectodermal Ubx BioID-interactomes.

(a) *Left panel:* Venn diagram representing the overlap of proteins enriched in close proximity to the wild-type (Ubx^{WT}) and mutant (Ubx^{N51A}) versions of Ubx protein in the neural system, which showed that 10 proteins interacted with Ubx preferentially on the chromatin (purple), 9 in the nucleus (dark purple) and 16 in the nucleoplasm (brown). *Right panel:* Fold enrichment of gene ontology terms of proteins representing the different overlap classes (chromatin, nucleus, nucleoplasm). **(b)** *Left panel:* Venn diagram representing the overlap of proteins enriched in close proximity to the wild-type (Ubx^{WT}) and mutant (Ubx^{N51A}) versions of Ubx protein in the neuroectodermal system, indicating that 68 proteins interacted with Ubx preferentially on the chromatin (purple), 10 in the nucleus (dark purple) and 38 in the nucleoplasm (brown). *Right panel:* Fold enrichment of gene ontology terms of proteins representing the different overlap classes (chromatin, nucleus, nucleoplasm). (p-value<0.05, for elav-chromatin maximal raw p-value 9.3E-05, elav-nucleus maximal raw p-value of 1.03E-04, sca-nucleus maximal raw p-value of 3.42E-03 and sca-nucleoplasm maximal raw p-value 1.26E-03 was used). **(c-d)** STRING-based reconstruction of interaction networks of all close-proximity partners of Ubx^{WT} in the neural system **(c)** and neuroectodermal tissue **(d)** identified by targeted BioID. Green circles represent RNA-binding/regulatory proteins, blue circles represent chromatin and DNA binding proteins, pink circles highlight TFs and white circles label proteins with unknown functions. GO term p-value are calculated with Fisher test and FDR correction. See also Supplementary Data 37-38.

Source files are provided in Source-Data file.

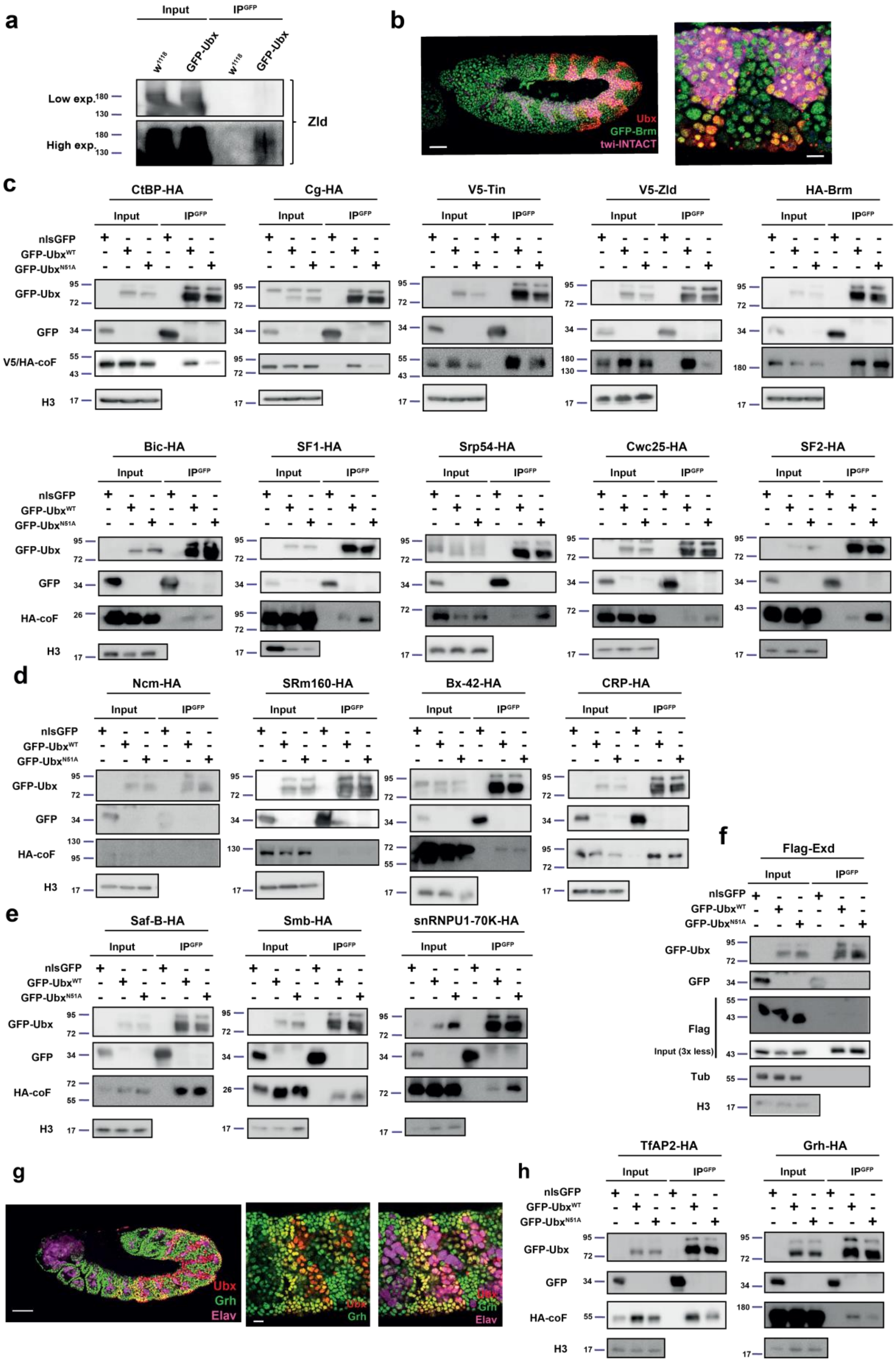

Supplementary Figure 6: Analysis of Ubx BioID-interactomes.

(a) Endogenous co-immunoprecipitation (co-IP) of Ubx close-proximity partners from nuclear extract of control (w^{1118}) or *GFP-Ubx* embryos, which carry a CRISPR/Cas9 engineered version of the *Ubx* gene, *GFP-Ubx*, at the endogenous locus[1]. The input fraction is indicated (lane 1-2). Zld (low and high exposure) co-immunoprecipitated with GFP-Ubx (IP^{GFP}-lane 4), which was not the case using purified extracts from w^{1118} embryos (IP^{GFP}-lane 3). **(b)** Immunostaining of stage 11 embryos (3-6 hours AEL) for Ubx (red) and the BioID identified mesodermal close-proximity partner Brm (MiMIC line) (green). To mark mesodermal cells, stainings were performed in the *twi-INTACT* background[2] (Supplementary Table 3b), which uses the tissue specific biotinylation of the nuclear membrane protein RanGAP, and allows the detection of mesodermal nuclei by streptavidin staining (magenta). Bottom panel represents a high-magnification image of mesodermal nuclei. **(c-f)** Co-IPs of HA- and V5-coupled Ubx close-proximity partners (CtBP, Cg, Tin, Zld, Brm, Bic, SF1, Srp54, Cwc25, SF2, Ncm, SRm160, Bx-42, Crp, Saf-B, Smb, snRNPU1-70K, referenced in Supplementary Data 39) and GFP fusion proteins (nls-GFP, GFP-Ubx^{WT}, GFP-Ubx^{N51A}), expressed in *Drosophila* S2R+ cells. Partners are detected in the immunoprecipitated fraction of GFP-Ubx^{WT} or GFP-Ubx^{N51A} (lane 5-6), while absent in the GFP negative control (lane 4), with the exception of Ncm. **(f)** Flag-Exd represents positive control of co-IP with GFP-Ubx^{WT} or Ubx^{N51A}. Tubulin (Tub) is a negative control. **(g)** Immunostaining of stage 11 embryos (3-6 hours AEL) for Ubx (red) and the neural partner Grh (green). The nervous system is highlighted by the pan-neural marker Elav (Magenta). **(h)** Co-immunoprecipitation of nlsGFP, GFP-Ubx^{WT}, GFP-Ubx^{N51A} expressed in S2R+ *Drosophila* cells with HA-coupled close-proximity neural partners (tfAP-2, Grh) as previously described. Partners are detected in the immunoprecipitated fraction of GFP-Ubx^{WT} or GFP-Ubx^{N51A} (lane 5-6), while absent in the GFP negative control (lane 4). Protein size is indicated relative to ladder position.

Images are representative of all embryos analysed per genotype over 2 sets of pooled embryos from independent collections.

Scale bar=50µm; Zoom scale bar=10µm. See Supplementary Data 39 for quantification.

Source files are provided in Source-Data file.

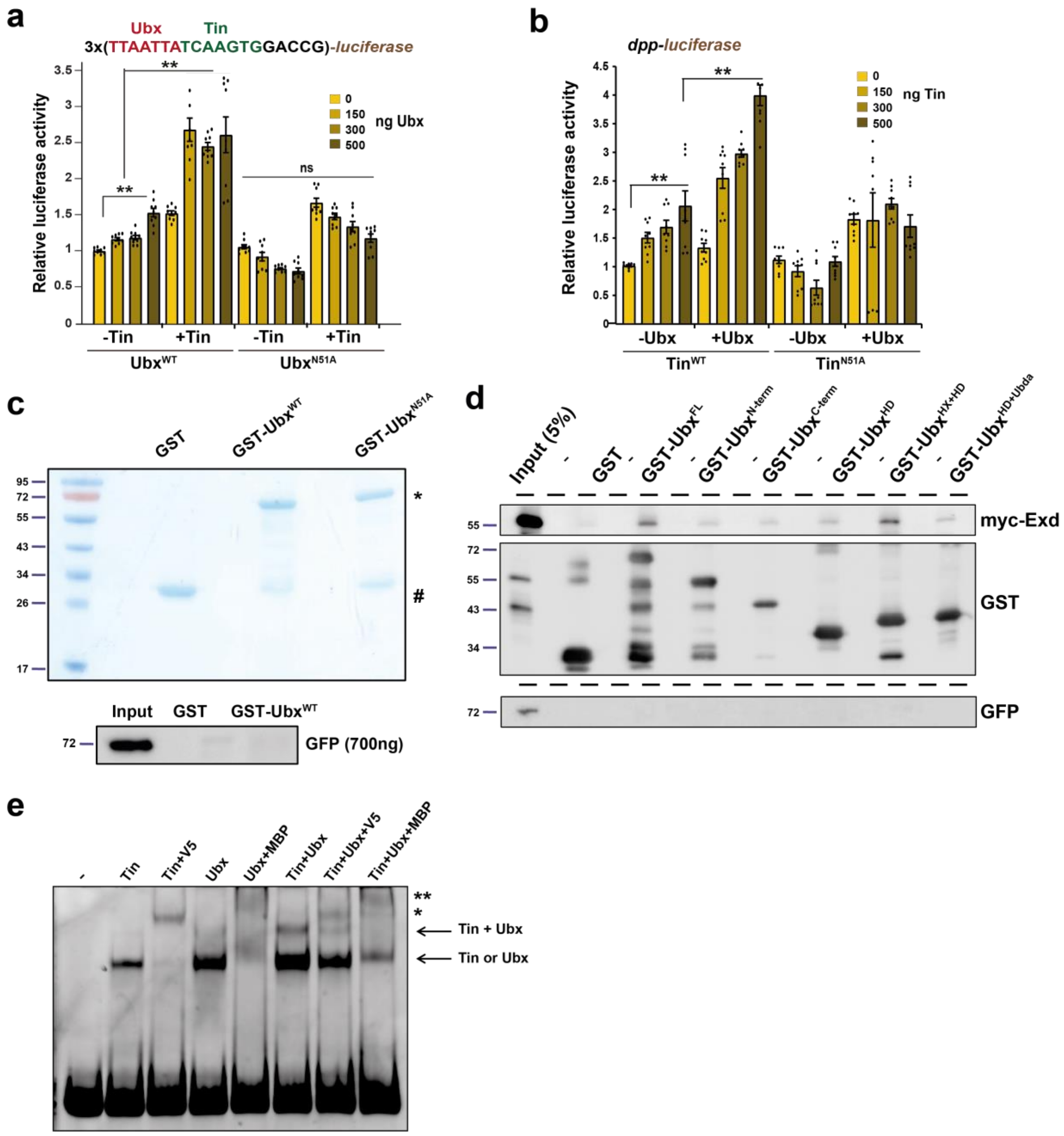

Supplementary Figure 7: The interaction between Ubx and Tin is direct and functional. **(a)** S2R+ cells were transfected with artificial enhancer containing multimerized 3x[Ubx-Tin] binding sites (red Ubx, green Tin) driving the expression of the luciferase together with the myc-Ubx and V5-Tin encoding plasmids. Increasing amounts of the different myc-Ubx plasmids were used in combination with the V5-Tin plasmid (100ng). **(b)** S2R+ cells were co-transfected with a *dpp674*-containing plasmid driving expression of the luciferase, myc-Ubx (100ng) and V5-Tin encoding plasmids. Increasing amounts of the Tin (Tin^{WT} or Tin^{N51A}) expressing plasmids were used. Transfection efficiency was normalized with Renilla activity originating from co-transfected pRT-TK or pActin-β-galactosidase plasmids. Results are indicated relative to the basal activity of the enhancer-luciferase plasmid. Graphics represent mean of three (n=3) independent experiments performed in triplicate with results expressed

as mean +/- sem and dot-plot (Anova p<0.01 **). **(c)** Expression of different GST-fused proteins as shown by Coomassie staining. Asterisk indicates Ubx and Rhomb the GST. **(d)** Pull-down assay using the indicated GST-fused Ubx derivatives and *in vitro* purified proteins, GST (negative control) and myc-Exd (positive control). Input is loaded as indicated. Protein size is indicated relative to ladder position. **(e)** EMSA with purified proteins (MBP-His-Ubx, His-V5-Tin) on fragment F3 of the dpp-674bp enhancer shows that Ubx and Tin bind specifically the fragment. Super-shift with V5 for Tin (star) and MBP for Ubx (double stars) antibodies are shown. Tin-Ubx combinatorial assays highlight DNA binding, both independently (arrow Tin or Ubx) and in complex (arrow Tin + Ubx). The presence of Tin and Ubx in complex is confirmed by the reduction of the ‘Tin + Ubx’ band (lane 6), shifted upon antibody addition (V5 and MBP, lane 7-8).
Source files are provided in Source-Data file.

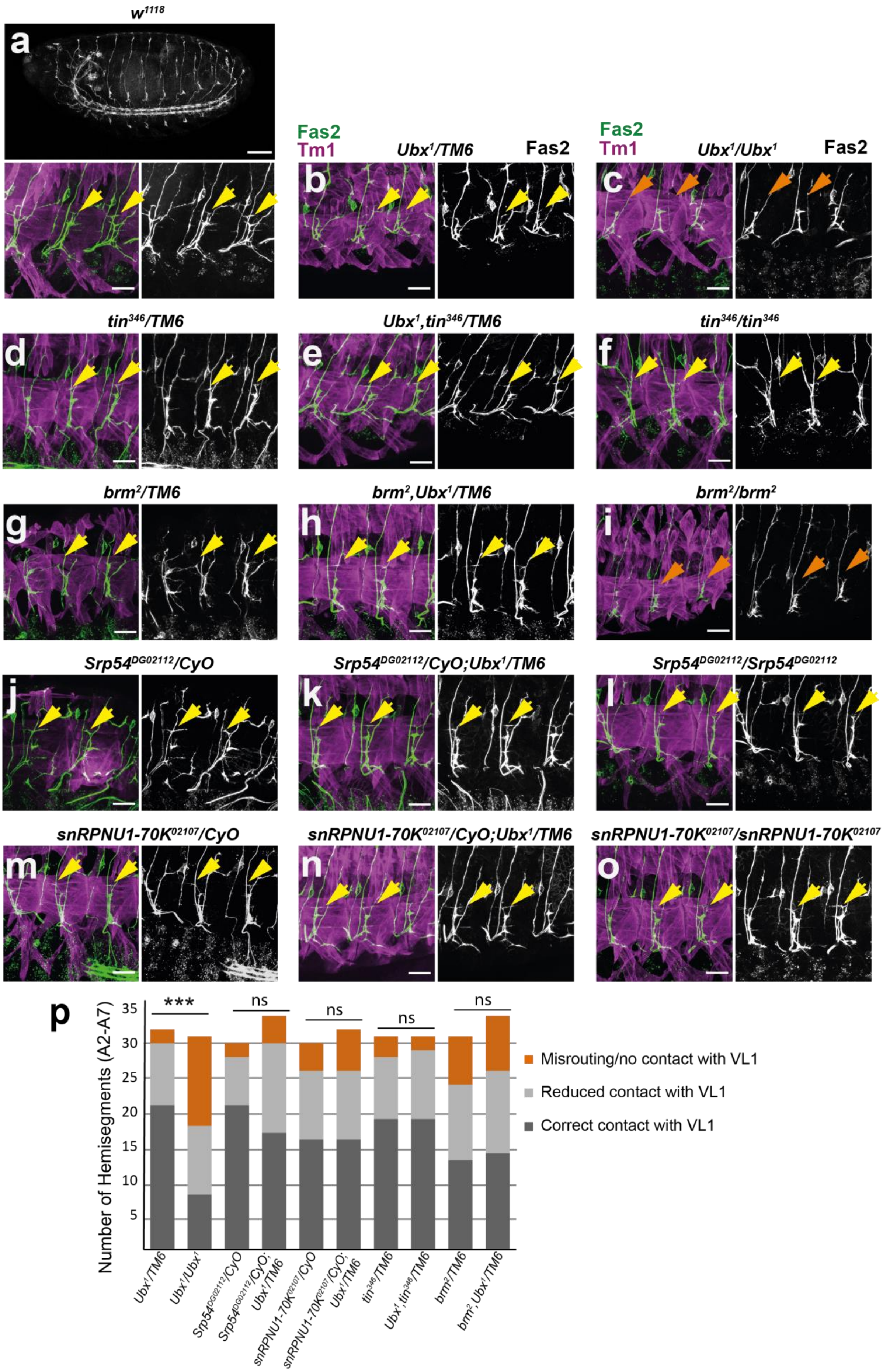

w^{1118}
a
Fas2
Tm1
$Ubx^{1}/TM6$
Fas2
b
Fas2
Tm1
Ubx^{1}/Ubx^{1}
Fas2
c
$tin^{346}/TM6$
d
$Ubx^{1},tin^{346}/TM6$
e
tin^{346}/tin^{346}
f
$brm^{2}/TM6$
g
$brm^{2},Ubx^{1}/TM6$
h
brm^{2}/brm^{2}
i
$Srp54^{DG02112}/CyO$
j
$Srp54^{DG02112}/CyO;Ubx^{1}/TM6$
k
$Srp54^{DG02112}/Srp54^{DG02112}$
l
$snRPNU1\text{-}70K^{02107}/CyO$
m
$snRPNU1\text{-}70K^{02107}/CyO;Ubx^{1}/TM6$
n
$snRPNU1\text{-}70K^{02107}/snRPNU1\text{-}70K^{02107}$
o
p
Number of Hemisegments (A2-A7)
35
30
25
20
15
10
5
***
ns
ns
ns
ns
Misrouting/no contact with VL1
Reduced contact with VL1
Correct contact with VL1
$Ubx^{1}/TM6$
Ubx^{1}/Ubx^{1}
$Srp54^{DG02112}/CyO$
$Srp54^{DG02112}/CyO;$ $Ubx^{1}/TM6$
$snRPNU1\text{-}70K^{02107}/CyO$
$snRPNU1\text{-}70K^{02107}/CyO;$ $Ubx^{1}/TM6$
$tin^{346}/TM6$
$Ubx^{1},tin^{346}/TM6$
$brm^{2}/TM6$
$brm^{2},Ubx^{1}/TM6$

Supplementary Figure 8: Lineage-specific functional cooperation of Ubx and broadly expressed BioID interactors on axon projections.

(a-o) Immunostainings of stage 17 embryos of the indicated genetic backgrounds with Fasciclin 2 (Fas2) to label axonal projections. Zooms of the ventral lateral muscle VL1 innervation of A2-A7 segments are presented with Tm1 in magenta and green, grey for Fas2. Images are representative of the majority (more than 60%) of 15 embryos analysed per genotype over pooled embryos from 3 independent collections, as quantified in **(p)**. Innervation of VL1 is highlighted with arrow heads, yellow for normal connexion and orange for altered connexion as illustrated for Ubx^{1} and brm^{2} homozygous mutants. **(p)** Quantification of VL1 innervation phenotype for heterozygous and double heterozygous (n=30 hemisegments or more) is presented and statistical analysis were performed with Chi^{2} test (p<0.001 ***), with a distinction between normal, reduced and misrouted or absent VL1 innervations. This showed a significant misrouting/loss of connexion of the innervation of the VL1 muscle for Ubx homozygous mutant compared to heterozygous embryos. Axonal patterns are statistically unchanged in double heterozygous mutants compared to single heterozygous mutant of Ubx or partners embryos.

Scale bar: 50µm for all embryos, 20µm for VL1 innervation presented pictures.

Raw files are presented in Source Data file.

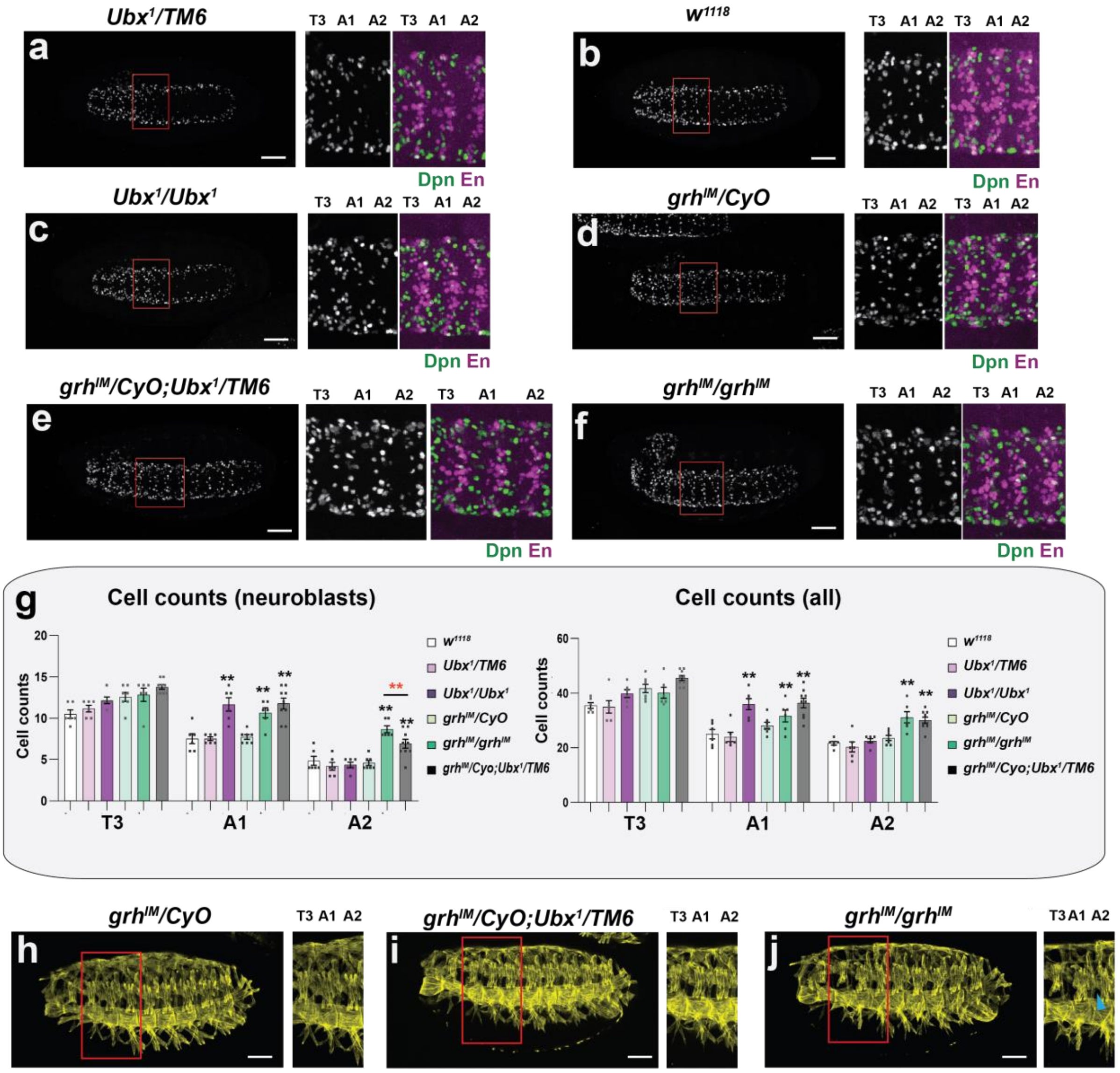

Supplementary Figure 9: Functional cooperation between Ubx and neural-specific interactor Grh.

(a-f) Deadpan (Dpn) immunostainings performed on stage 17 embryos of the indicated genetic backgrounds label neural cells and neuroblasts (NBs) of the Ventral Nerve Chord (VNC). Red boxes highlight thoracic T3, abdominal A1 and A2 segments, which are shown as magnifications (right side of image). Dpn (white, green) highlights the NBs, Engrailed (En, purple) labels the anterior region of each parasegment. Maximum Z-projections of ventral view are presented. Images are representative of all embryos analysed (n=6 or more) per genotype over 2 sets of pooled embryos from independent collection as quantified in **(g)**. **(g)** Summary of phenotypes is illustrated by the quantifications of NBs (inside part of the VNC) and total cells numbers per segment (n=6 embryos or more). Graphics represent mean and dot-plot +- sem (Anova p<0.01 ** compared to *Ubx^1/TM6*, red star compares *grh^{IM}/grh^{IM}* and *grh^{IM}/CyO;Ubx^1/TM6*). Ubx and Grh genetically interact for the regulation of NBs number

during programmed cell death[4-6]. **(h-j)** Muscle pattern visualized by Tropomyosin (Tm1) immunostainings of stage 16 embryos of the indicated genetic backgrounds. Red boxes highlight the thoracic T3 and the abdominal A1 and A2 segments, which are shown as magnifications (right side). Maximum Z-projections of lateral view are presented. Single and double heterozygous mutants (h-i) did not display any muscle phenotype. Single homozygous grh^{IM} mutants (j) presented alteration of A2 segment muscle morphology (50% penetrance). For the muscle pattern, images are representative of all embryos or half *for* grh^{IM} mutant (n=50 observed), over 2 sets of pooled embryos from independent collection.
Scale bar: 50µm.
Source files are provided in Source-Data file.

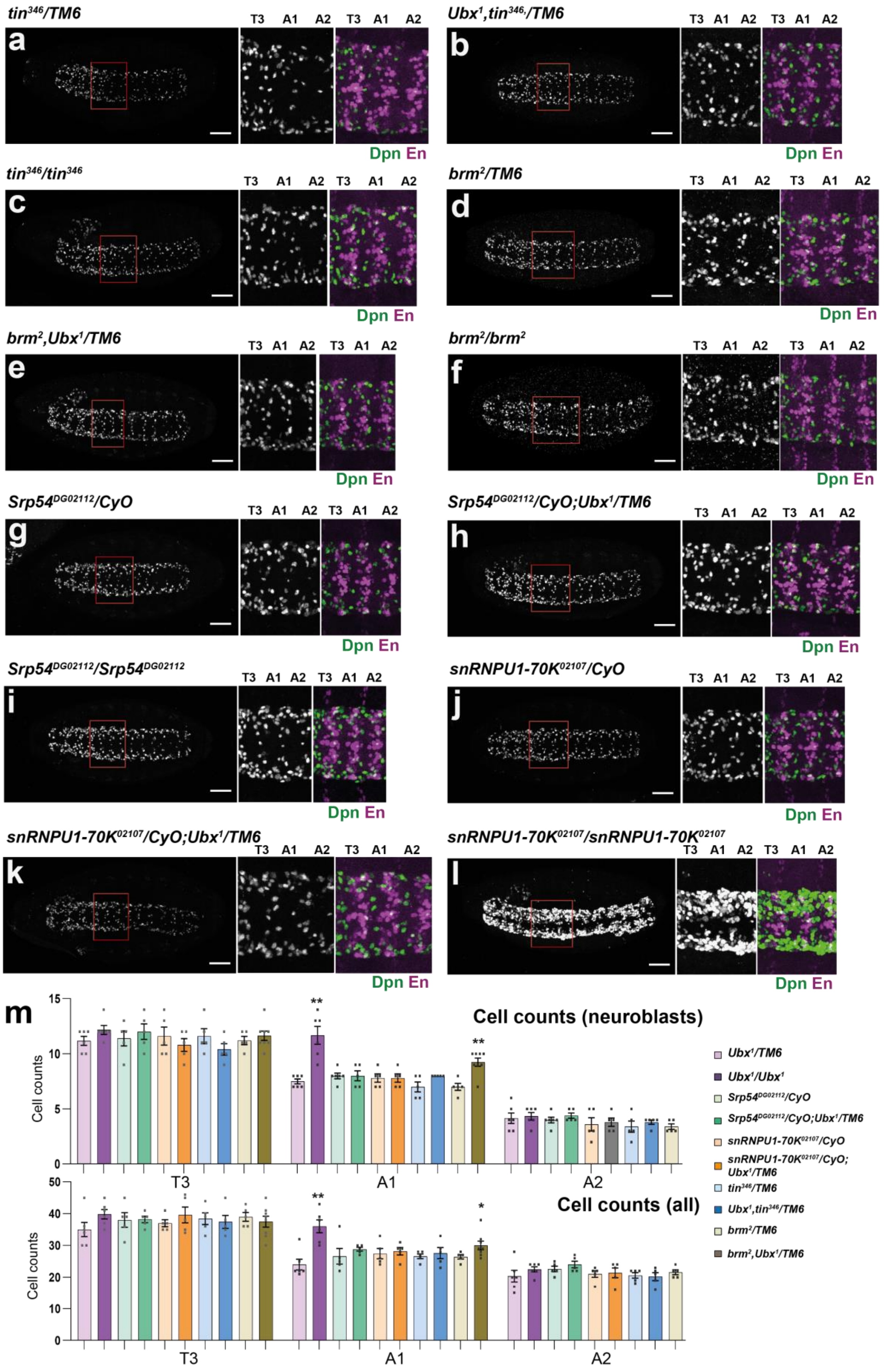

Supplementary Figure 10: Functional cooperation of Ubx and broadly expressed BioID interactors on neuroblasts number.

(a-l) Deadpan (Dpn) immunostainings performed on stage 17 embryos of the indicated genetic backgrounds label neural cells and neuroblasts of the VNC. Red boxes highlight the thoracic segment T3 and the abdominal segments A1 and A2, which are shown as magnifications on the right side of each image, presenting Dpn (white, green) and Engrailed (En, purple) labelling the segment identity. Maximum Z-projections of ventral view are presented. Images are representative of all embryos analysed (n=5 or more) per genotype over 2 sets of pooled embryos from independent collection as quantified in **(m)**. **(m)** Summary of phenotypes is illustrated by the quantifications of NBs (inside part of the VNC) and total cells numbers per segment (n=5 embryos or more). Graphic represent mean and dot-plot +- sem (Anova $p<0.05$*, $p<0.01$ ** compared to *Ubx*^{1}*/TM6*). Genetic interaction has been observed between Brm and Ubx, characterized by 1-2 additional neuroblasts in the A1 segment, while Ubx homozygous mutant present 4 additional neuroblasts in A1 segment. Only snRNPU1-70K single mutant (l) exhibit a strong phenotype characterized by an increase level of Dpn expression and a strong alteration of Dpn-positive cell shape (triangular and bigger). No genetic interaction has been observed between Ubx and the mesoderm-specific cofactors (Srp54, snRNPU1-70K, Tin) confirming the suitability of the BioID to study lineage-specific functional interactive networks.

Scale bar: 50µm.

Raw files are provided in Source-Data file.

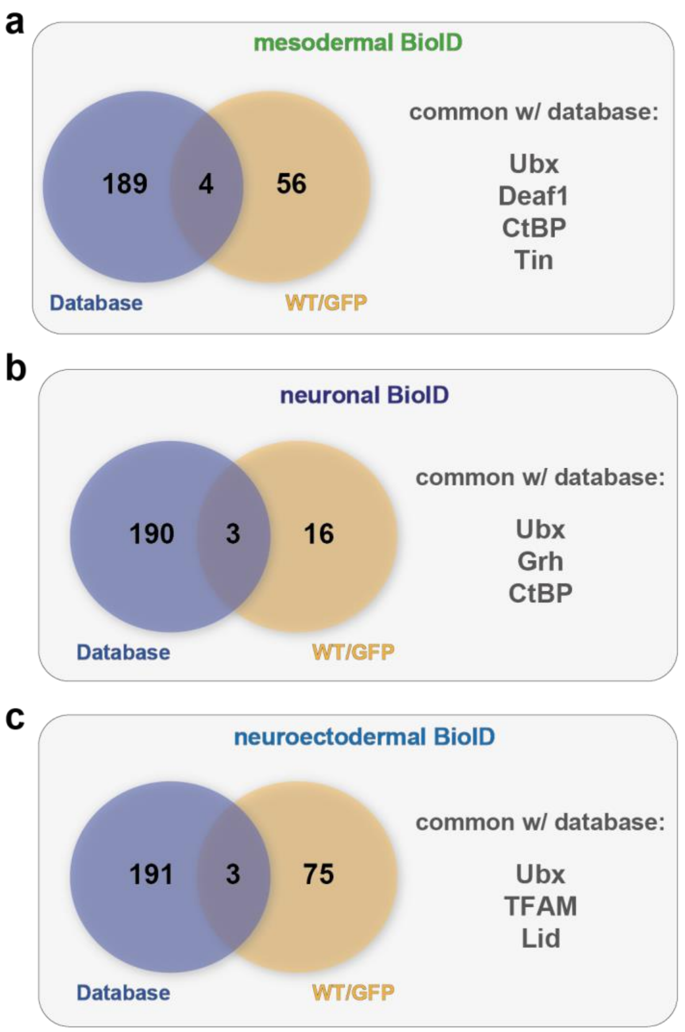

Supplementary Figure 11: Comparison of Ubx interaction partners identified in targeted BioID with known interactors deposited in databases.

(a, b, c) Venn diagrams representing the overlaps of proteins interacting with Ubx, with proteins which have been identified previously and are deposited in databases (Flybase, BioGrid, DroID) shown in blue, and proteins identified by targeted BioID in this study shown in brown. Overlaps are shown individually for the Ubx^{WT} mesodermal BioID (a), neural BioID (b) and neuroectodermal BioID (c). The names of the commonly identified proteins are shown on the right.

SUPPLEMENTARY TABLES

Twist-BioID	**WT replicate (R)**			
	**WT/GFP_R1**	**WT/GFP_R2**	**WT/GFP_R3**	**WT/GFP_R4**
Median	-0.097015744	-0.103096254	0.668873576	-0.184423657
Average	0.012615692	0.184383968	0.774421427	-0.06637495
5th percentile	-1.352908323	-1.504342018	-0.936212638	-1.732151799
95th percentile	1.65876915	2.8506038	2.778163663	2.01929148
Median 90%	-0.090027492	-0.121454445	0.668873576	-0.184423657
Average 90%	-0.064351407	0.021183971	0.732923885	-0.096419544
SD 90%	0.611388705	1.140420207	0.908634602	0.847782738
**Mean+2SD (stat sign)**	**1.158426003**	**2.302024385**	**2.55019309**	**1.599145932**
CI -2SD	-1.287128817	-2.259656443	-1.08434532	-1.79198502
	**N51A replicate (R)**			
	**N51A/GFP_R1**	**N51A/GFP_R2**	**N51A/GFP_R3**	**N51A/GFP_R4**
Median	0.020276733	-0.1336044	0.982520998	-0.377984615
Average	0.11048024	0.031197602	0.945495043	-0.34169657
5th percentile	-1.109208547	-1.568170653	-1.062616409	-2.116069643
95th percentile	1.517482064	1.937298129	2.970252623	1.897588285
Median 90%	0.020276733	-0.138847137	0.982520998	-0.377984615
Average 90%	0.049197504	-0.084028447	0.92513137	-0.373504658
SD 90%	0.524783216	0.827026607	0.933855647	0.836994435
**Mean+2SD (stat sign)**	**1.098763935**	**1.570024768**	**2.792842664**	**1.300484212**
CI -2SD	-1.000368928	-1.738081662	-0.942579925	-2.047493529

Supplementary Table 1a: Summary of parameters set for significant enrichment of proteins in for twist-BioID and related to Supplementary Data 4-11. CI is confidence interval.

Elav-BioID	**WT replicate (R)**			
	**WT/GFP_R1**	**WT/GFP_R2**	**WT/GFP_R3**	**WT/GFP_R4**
Median	-0.341800173	0.214320663	-0.682579072	-0.099613229
Average	-0.156546034	0.409956622	-0.488187125	-0.086507902
5th percentile	-1.092827297	-0.811946543	-2.172750369	-1.576129811
95th percentile	1.831542541	2.041729722	1.770072629	1.771628898
Median 90%	-0.341800173	0.214320663	-0.682579072	-0.099613229
Average 90%	-0.233209596	0.358794642	-0.525906387	-0.107531923
SD 90%	0.535316854	0.672582139	0.859868078	0.83008713
**Mean+2SD (stat sign)**	**0.837424112**	**1.703958919**	**1.19382977**	**1.552642338**
CI -2SD	-1.303843305	-0.986369635	-2.245642544	-1.767706183

	N51A replicate (R)			
	**N51A/GFP_R1**	**N51A/GFP_R2**	**N51A/GFP_R3**	**N51A/GFP_R4**
Median	-0.04492285	-0.08493424	-0.208302009	-0.008072329
Average	-0.122564594	-0.004885735	-0.182167405	0.027061745
5th percentile	-2.035221937	-1.869095354	-1.755592943	-1.483823843
95th percentile	1.766218143	1.871383282	1.313364756	1.363345432
Median 90%	-0.04492285	-0.08493424	-0.227316665	-0.008072329
Average 90%	-0.151362555	-0.045244173	-0.153906141	0.0126169
SD 90%	0.828328919	0.811142659	0.761393684	0.709333973
**Mean+2SD (stat sign)**	**1.505295282**	**1.577041145**	**1.368881226**	**1.431284847**
CI -2SD	-1.808020393	-1.667529491	-1.676693508	-1.406051047

Supplementary Table 1b: Summary of parameters set for significant enrichment of proteins in for elav-BioID and related to supplementary Data 12-19. CI is confidence interval.

Scabrous-BioID	**WT replicate (R)**			
	**WT/GFP_R1**	**WT/GFP_R2**	**WT/GFP_R3**	**WT/GFP_R4**
Median	0.148130575	0.145926053	-0.151080444	0
Average	-0.170389043	-0.054192403	-0.187309852	-0.003920705
5th percentile	-3.063801048	-2.89967225	-1.51038349	-1.238393232
95th percentile	1.365363586	1.677523382	1.199575011	1.161359971
Median 90%	0.150560125	0.145926053	-0.151080444	0
Average 90%	-0.065562701	0.061218048	-0.151782418	0.028675633
SD 90%	0.950156725	0.878427944	0.638836779	0.567949488
**Mean+2SD (stat sign)**	**1.834750749**	**1.818073937**	**1.12589114**	**1.164574609**
CI -2SD	-1.965876151	-1.695637841	-1.429455976	-1.107223343
	**N51A replicate (R)**			
	**N51A/GFP_R1**	**N51A/GFP_R2**	**N51A/GFP_R3**	**N51A/GFP_R4**
Median	-0.035430535	-0.065372344	-0.067013614	0.027447418
Average	-0.166619876	-0.2319259	0.029168785	0.141745565
5th percentile	-1.93266277	-2.499683062	-1.920726618	-0.812961326
95th percentile	1.157431707	1.317254639	2.236838895	1.239663922
Median 90%	-0.035430535	-0.065372344	-0.067013614	0.027447418
Average 90%	-0.104392326	-0.152976778	0.003154237	0.125254275
SD 90%	0.706942877	0.768245414	0.941302717	0.458358358
**Mean+2SD (stat sign)**	**1.309493428**	**1.383514049**	**1.885759671**	**1.041970991**
CI -2SD	-1.51827808	-1.689467606	-1.879451196	-0.791462442

Supplementary Table 1c: Summary of parameters set for significant enrichment of proteins in for scabrous-BioID and related to supplementary Data 20-27. CI is confidence interval.

	**Fold enrichment (relative to profile)**			
**GO term Biological process**	**Tin/Ubx expressed**	**Tin/Ubx**	**Ubx**	**Tin**
dorsal vessel heart proper cell fate commitment	49.17	46.67	0	0
ectoderm and mesoderm interaction	49.17	46.67	0	0
embryonic heart tube anterior/posterior pattern specification	49.17	46.67	0	0
positive regulation of apoptotic process involved in morphogenesis	49.17	46.67	0	0
gonadal mesoderm development	36.88	35	0	6.19
muscle cell fate specification	35.12	33.33	0	7.07
muscle cell fate commitment	31.61	30	3.55	7.07
mesodermal cell fate specification	30.26	28.72	0	5.44
mesodermal cell fate determination	29.5	28	0	0
muscle cell fate determination	29.5	28	0	7.07
cardioblast differentiation	22.69	21.54	3.82	5.98
cardiac cell fate commitment	22.35	21.21	0	6.43
mesodermal cell differentiation	21.07	20	2.96	5.39
mesodermal cell fate commitment	21.07	20	2.96	5.39
positive regulation of muscle organ development	21.07	20	0	0
cell fate commitment involved in formation of primary germ layer	20.8	19.74	0	5.44
pericardial nephrocyte differentiation	19.67	18.67	0	5.66
muscle fiber development	18.91	21.54	0	4.35
cell fate specification involved in pattern specification	18.44	17.5	0	7.07
regulation of muscle organ development	18.44	17.5	0	5.75
regulation of muscle tissue development	18.44	17.5	0	5.3
regulation of striated muscle tissue development	18.44	17.5	0	5.3
cardiocyte differentiation	17.88	16.97	3.2	6.11
mesoderm formation	16.9	16.04	2.98	5.75
mesoderm morphogenesis	15.45	14.67	2.84	5.86
cardiac muscle tissue development	14.75	14	0	0
mesoderm migration involved in gastrulation	13.41	12.73	0	5.79
muscle tissue development	13.24	12.56	3.03	3.26
larval somatic muscle development	10.76	10.21	0	3.98
mesoderm development	9.74	9.24	2.38	3.71
heart development	9.64	9.15	2.39	3.95
circulatory system development	9.37	8.89	0	3.84
muscle cell cellular homeostasis	8.48	8.05	0	0

muscle organ development	6.86	6.51	2.79	3.78
muscle structure development	6.86	6.95	0	3.49

Supplementary Table 2a: Fold enrichment of gene ontology terms related to mesodermal function of genes bound by Ubx (mesoderm ChIP-seq from[3]), Tin (ChIP-on-ChIP from[9]), Ubx/Tin, and Ubx/Tin specifically expressed in the mesoderm according to the mesodermal transcriptome from Domsch et al, (2019)[3].

	**Fold enrichment (relative to profile)**			
**GO term Biological process**	**Tin/Ubx expressed**	**Tin/Ubx**	**Ubx**	**Tin**
endoderm formation	35.12	33.33	0	7.07
head segmentation	24.58	23.33	5.31	6.75
mesenchyme development	19.67	18.67	0	5.66
formation of primary germ layer	18.91	17.95	9.42	5.8
regulation of cell fate specification	16.39	15.56	6.52	6.29
haltere development	15.13	14.36	3.14	5.44
neuroblast fate commitment	14.75	14	7.25	6.13
germ cell migration	14.34	13.61	11.59	3.54
neuroblast differentiation	14.2	13.48	10.87	5.81
stem cell differentiation	13.47	12.79	17.63	5.42
ectoderm development	11.87	11.26	7	4.88
reproductive system development	11.85	11.81	20.05	4.43
stem cell development	9.46	8.97	6.28	5.71
neuron fate commitment	9	9.2	17.15	4.38

Supplementary Table 2b: Fold enrichment of gene ontology terms related to general function of genes bound by Ubx (mesoderm ChIP-seq from[3]), Tin (ChIP on ChIP from[9]), Ubx/Tin, and Ubx/Tin specifically expressed in the mesoderm according to the mesodermal transcriptome from Domsch et al, (2019)[3].

	**Fold enrichment (relative to profile)**				
**GO term Biological process**	**Grh/Ubx expressed**	**Grh/Ubx no exp.**	**Grh/Ubx**	**Ubx**	**Grh**
axon development	2.93	3.87	3.47	2.17	2.11
axon guidance	3.14	4.24	3.77	2.19	2.13
axonogenesis	3.03	4.01	3.59	2.23	2.09
cell morphogenesis involved in neuron differentiation	2.76	3.3	3.07	2.01	2
cell projection morphogenesis	2.73	3.48	3.16	2.05	2
chemotaxis	3.12	4.24	3.76	2.14	2.14
larval development	4.13	3.69	3.88	1.73	2.05

maintenance of location	6.31	0	3.49	0	0
neuron development	2.41	2.85	2.66	1.82	1.95
neuron differentiation	2.24	2.8	2.55	1.82	2
neuron projection development	2.75	3.12	2.96	1.99	2.02
neuron projection guidance	3.04	4.1	3.64	2.16	2.12
neuron projection morphogenesis	2.76	3.29	3.06	2.04	1.99
segmentation	3.48	4.69	4.17	1.94	1.99
tube development	2.69	4.06	3.47	1.83	2.16

Supplementary Table 2c: Fold enrichment of gene ontology terms related to neural function of genes bound by Ubx (neural ChIP-seq from[3]), Grh (ChIP-seq from[10]), Ubx/Grh and Ubx/Grh specifically expressed and non-expressed (*no exp.)* in the neural system according to the transcriptome from Domsch et al, (2019)[1].

	**Fold enrichment (relative to profile)**				
**GO term Biological process**	**Grh/Ubx expressed**	**Grh/Ubx no exp.**	**Grh/Ubx**	**Ubx**	**Grh**
anterior/posterior lineage restriction, imaginal disc	0	33.2	18.91	0	0
regulation of striated muscle contraction	0	33.2	18.91	0	0
specification of animal organ identity	0	33.2	18.91	0	0
head morphogenesis	0	26.56	0	0	0
notum cell fate specification	0	26.56	0	0	0
muscle cell fate specification	0	25.3	14.41	0	0
Bolwig's organ morphogenesis	0	19.67	11.21	0	0
larval visceral muscle development	0	19.67	11.21	0	0
cardioblast cell fate commitment	0	18.97	0	0	0
muscle cell fate commitment	0	18.97	10.81	0	0
cardiac cell fate commitment	0	16.1	9.17	0	0
visceral muscle development	0	14.76	8.4	0	0
cardioblast differentiation	0	13.62	0	0	0
nephrocyte differentiation	0	13.62	0	0	0
regulation of muscle system process	0	11.8	0	0	0
cardiocyte differentiation	0	10.06	0	0	2.71
mesodermal cell migration	0	8.85	0	0	0
mesodermal cell differentiation	0	8.43	0	0	2.83
mesodermal cell fate commitment	0	8.43	0	0	2.83

midgut development	0	7.59	5.04	0	2.51

Supplementary Table 2d: Fold enrichment of gene ontology terms specifically enriched for genes bound by Ubx (neural ChIP-seq from[3]), Grh (ChIP-seq from[10]), Ubx/Grh and Ubx/Grh expressed and non-expressed (*no exp.)* in the neural system according to the transcriptome from Domsch et al, (2019)[3]. The GO terms presented are specifically enriched in the Ubx/Grh bound and non-expressed gene fraction.

Plasmid List	Source	Reference	additional information
pActin-Gal4	Lohmann lab	-	
pUAST-myc-BirA* #1	Generated by cloning	-	
pUAST-myc-BirA*-UbxIa #1	Generated by cloning	-	
pUAST-attB-empty	Lohmann lab	-	
pCDNA3.1-BioID1	Addgene	#35700	A promiscuous biotin ligase fusion protein identifies proximal and interacting proteins in mammalian cells. Roux KJ, Kim DI, Raida M, Burke B. J Cell Biol. 2012 Mar 12. 10.1083/jcb.201112098 PubMed 22412018
pUAST-attB-myc-Exd	Generated by cloning	-	
pUAST-myc-BirA*-GFP-nls	Generated by cloning	-	
pUAST-attB-myc-BirA*-Ubx N51A	Generated by cloning	-	
pUAST-attB-myc-BirA*-Ubx-Comb	Generated by cloning	-	
pUAST-attB-flag-Exd	Generated by cloning	-	
pET-His-MBP1a-GFP	Generated by cloning	-	
pET-His-MBP1a-mBU WT	Generated by cloning	-	
pET-His-MBP1a-mBU N51A	Generated by cloning	-	
pCDNA3.1-BioID2-MCS	Addgene	#74223	An improved smaller biotin ligase for BioID proximity labeling. Kim DI, Jensen SC, Noble KA, Kc B, Roux KH, Motamedchaboki K, Roux KJ. Mol Biol Cell. 2016 Feb 24. pii: mbc.E15-12-0844. 10.1091/mbc.E15-12-0844 PubMed 26912792
pUAST-attB-BioID2	Generated by cloning	-	
pUAST-attB-BioID2-Ubx WT	Generated by cloning	-	
pUAST-attB-BioID2-Ubx N51A	Generated by cloning	-	
pUAST-attB-BioID2-Ubx Comb	Generated by cloning	-	
pET-His-myc-Exd	Generated by cloning	-	
pUAST-attB-HA-Brm FL	Generated by cloning	-	
pUAST-attB-myc-GG-Ubx FL WT	Generated by cloning	-	

pUAST-attB-myc-GG-Ubx FL N51A	Generated by cloning	-	
pUAST-attB-GFP-GG-Ubx FL WT	Generated by cloning	-	
pUAST-attB-GFP-GG-Ubx FL N51A	Generated by cloning	-	
pGL3-min-dpp674-luciferase	From Ana Rogulja-Ortmann	-	
CtBP	DGRC	UFO01125	
Saf-B	DGRC	FMO02988	
Bx-42	DGRC	UFO02028	
Bic	DGRC	UFO01314	
Cg	DGRC	UFO07207	
CRP	DGRC	UFO09755	
pUAST-V5-Zelda	Generated by cloning from FI17950	cDNA from DGRC	
Cwc25	DGRC	UFO07445	
grh	DGRC	UFO07467	
TfAP-2	DGRC	FMO01306	
snRNP-U1-70K	DGRC	UFO06121	
Srp54	DGRC	UFO06136	
SRm160	DGRC	UFO10126	
SF2	DGRC	UFO02920	
pUAST-attB-V5-Tinman-WT	Generated by cloning	-	
pUAST-attB-V5-Tinman-N51A	Generated by cloning	-	
pUAST-attB-nlsGFP	Generated by cloning	-	
SF1	DGRC	UFO08918	
pRT-TKrenilla	Promega	E2241	
pGEX-2P-6-GST	Lohmann lab	-	
pGEX-Ubx-WT	Generated by cloning	-	
pGEX-Ubx-N51A	Generated by cloning	-	
pGEM-dpp (cDNA)	Generated by cloning	-	
pET-His-V5-Tinman	Generated by cloning	-	
pGEX-Ubx-Nterm	Generated by cloning	-	
pGEX-Ubx-Cterm	Generated by cloning	-	
pGEX-Ubx-HD	Generated by cloning	-	
pGEX-Ubx-HX+HD	Generated by cloning	-	
pGEX-Ubx-HD+UbdA	Generated by cloning	-	
pGL3-min-3x[Ubx-Tin]-luciferase	Generated by cloning	-	
pActin-Beta-Galactosidase	DGRC	#1220	

Supplementary Table 3a: List of plasmids

**fly lines**	**reference**	**citation**
w1118		
UAS-myc-BirA-nlsGFP*	Generated by cloning	-
UAS-myc-BirA-Ubx^{WT}*	Generated by cloning	-
UAS-myc-BirA-Ubx^{N51A}*	Generated by cloning	-

GFP-Ubx crisPR	Domsch et al., 2019	Domsch, K. et al. The Hox transcription factor Ubx stabilizes lineage commitment by suppressing cellular plasticity in Drosophila. eLife 8, (2019).
twist-Gal4	Baker et al., 1996	Baker, R. & Schubiger, G. Autonomous and nonautonomous Notch functions for embryonic muscle and epidermis development in Drosophila. Dev. Camb. Engl. 122, 617–626 (1996).
elav-Gal4	Yao et al., 1994	Yao, K. M. & White, K. Neural specificity of elav expression: defining a Drosophila promoter for directing expression to the nervous system. J. Neurochem. 63, 41–51 (1994).
scabrous-Gal4	Hessinger et al., 2017	Hessinger, C., Technau, G. M. & Rogulja-Ortmann, A. The Drosophila Hox gene Ultrabithorax acts in both muscles and motoneurons to orchestrate formation of specific neuromuscular connections. Development 144, 139–150 (2017).
armadillo-Gal4	Sanson et al., 1996	Sanson, B., White, P. & Vincent, J.-P. Uncoupling cadherin-based adhesion from wingless signalling in Drosophila. *Nature* **383**, 627–630 (1996).
twi-INTACT	Steiner et al., 2012	Steiner, F. A., Talbert, P. B., Kasinathan, S., Deal, R. B. & Henikoff, S. Cell-type-specific nuclei purification from whole animals for genome-wide expression and chromatin profiling. Genome Res. 22, 766–777 (2012).
twi-INTACT;MIMIC-GFP-Brm	Generated by crossing from MIMIC-line	Venken, K. J. T. et al. MiMIC: a highly versatile transposon insertion resource for engineering Drosophila melanogaster genes. Nat. Methods 8, 737–743 (2011).
Ubx^{1}*/TM6,Dfd>LacZ*	Generated by crossing from bloomington stock	Grell, R. F. Non Random Assortment of Non-Homologous Chromosomes in Drosophila Melanogaster. Genetics 44, 421–435 (1959).
tin^{346}*/TM6,Dfd>LacZ*	Generated by crossing from bloomington stock	Azpiazu, N. & Frasch, M. tinman and bagpipe: two homeo box genes that determine cell fates in the dorsal mesoderm of Drosophila. Genes Dev. 7, 1325–1340 (1993).
brm^{2}*/TM6-Dfd>lacZ*	Generated by crossing from bloomington stock	Kennison, J. A. & Tamkun, J. W. Dosage-dependent modifiers of polycomb and antennapedia mutations in Drosophila. Proc. Natl. Acad. Sci. 85, 8136–8140 (1988).
Srp54^{DG02112}*/CyO-wg>lacZ*	Generated by crossing from bloomington stock	Huet, F. et al. A deletion-generator compound element allows deletion saturation analysis for genomewide phenotypic annotation. Proc. Natl. Acad. Sci. 99, 9948–9953 (2002).
snRNPU1-70K^{02107}*/CyO-wg>lacZ*	Generated by crossing from bloomington stock	Spradling, A. C. et al. The Berkeley Drosophila Genome Project gene disruption project: Single P-element insertions mutating 25% of vital Drosophila genes. Genetics 153, 135–177 (1999).
Ubx^{1}*,tin*^{346}*/TM6,Dfd>LacZ*	Generated by crossing	-
brm^{2}*,Ubx*^{1}*/TM6-Dfd>lacZ*	Generated by crossing	-
Srp54^{DG02112}*/CyO-wg>lacZ;Ubx*^{1}*/TM6-Dfd>lacZ*	Generated by crossing	-
snRNPU1-70K^{02107}*/CyO-wg>lacZ;Ubx*^{1}*/TM6-Dfd>lacZ*	Generated by crossing	-
Grh^{IM}*/CyO-wg>lacZ*	Generated by crossing from bloomington stock	Ostrowski, S., Dierick, H. A. & Bejsovec, A. Genetic control of cuticle formation during embryonic development of Drosophila melanogaster. Genetics 161, 171–182 (2002).
Grh^{IM}*/CyO-wg>lacZ;Ubx*^{1}*/TM6-Dfd>lacZ*	Generated by crossing	-

Supplementary Table 3b: List of fly lines

Oligonucleotides	sequences	information
f-myc-BirA*	ACTGAATCAAAATGGAACAAAAACTCATCTCAGAAG	cloning of pUAST-attB-myc-BirA*
r-myc-BirA*	CGTAGGATCTCATATGACCCCGCCTGAACCTCCCTTCTCTGCGCTTCTCAGG	cloning of pUAST-attB-myc-BirA*
f-Ubx	GGACATATGAACTCGTACTTTGAACAGG	cloning of pUAST-attB-myc-BirA*-Ubx WT
r-Ubx	AAATCTAGAGCTACTGATCTAAGTGTC	cloning of pUAST-attB-myc-BirA*-Ubx WT
f-Ubx-N51A	CTGGTTCCAGGCCCGGCGAATG	cloning of pUAST-attB-myc-BirA*-Ubx N51A
r-Ubx-N51A	ATCTTGATCTGCCGCTCC	cloning of pUAST-attB-myc-BirA*-Ubx N51A
f-Ubx-Comb	GCCCGGCGAGCGAAGCTGAAGAAGGAGATCCAG	cloning of pUAST-attB-myc-BirA*-Ubx Comb
r-Ubx-Comb	CGCGAACCAGGCCTTGATCTGCCGCTCCGT	cloning of pUAST-attB-myc-BirA*-Ubx Comb
f-GFP-nls	GAAAGATCTATGAGTAAAGGAGAAGAACTTTTCACTGGAG	cloning GFP-nls in pUAST-attB-/myc-BirA*
r-GFP-nls	CTTTCTAGATCACACCTTCCTCTTCTTCTTGGGGAATTCTTTGTATAGTTCATCCATGCCAT	cloning GFP-nls
f-flag-Exd	GAAAGATCTATGGATTACAAGGATGACGACGATAAGAATTCAAGGATGGAGGACCCCAATCGCAT	cloning pUAST-attB-flag-Exd
r-Exd	AAACTCTAGAGTCAGGGGCTTAGATCCTGAT	cloning pUAST-attB-flag-Exd
f2-myc-BirA*	AAACCATGGATGGAACAAAAACTCATCTC	cloning pET-myc-BirA-Ubx
r2-Ubx	CCTAAGCTTGCTACTGATCTAAGTGTCCA	cloning pET-myc-BirA-Ubx
f-BioID2	ACTGAATTCAAAATGGAACAAAAACTCATCTCA	cloning bioID2 in pUAST-attB-X-Ubx/GFP
r-BioID2	CGTAGATCTCATATGACCGCCTGAACCTCCGCTTCTTCTCAGGCTGA	cloning bioID2 in pUAST-attB-X-Ubx/GFP
f2-Ubx	AAACCATGGAAATGAACTCGTACTTTGAACA	cloning pET-Ubx FL with r2-Ubx
f-myc-Ubx	AGGAGGGGTACCTCAAAATGGAGCAGAAACTCATCTCTGAAGAGGATCTGGGAGGAATGAACTCGTACTTTGAACAGG	cloning of pUAST-attB-myc-Ubx with r-Ubx
f-HA-Brm	AACAACGCGGCCGCTCAAAATGTACCCATACGATGTTCCAGATTACGCTATGGCCTCGCCCTCTCC	cloning of pUAST-attB-HA-Brm
r-Brm	AACAACTCTAGACCCCTAGTCCATGTCATCGTCGTCATCGTCTGAAA	cloning of pUAST-attB-HA-Brm
f-GFP	AACAACGAATTCTCAAAATGAGTAAAGGAGAAGAACTTTTCACTGGAG	cloning of pUAST-GFP-Ubx
r-GFP	CCGCCTGAACCTCCTTTGTATAGTTCATCCATGCCATGTGTAATCC	cloning of pUAST-GFP-Ubx
f-V5-Zld	AACAACGAATTCTCAAAATGGGTAAGCCTATCCCTAACCCTCTCCTCGGTCTCGATTCTACGATGACGAGCATTAAGACCGA	cloning pUAST-attB-V5-Zld
r-Zld	TGGTGGTCTAGACTCAGTAGAGCTCTATGCTCTT	cloning pUAST-attB-V5-Zld
f-V5-Tin	AACAACGAATTCTCAAAATGGGTAAGCCTATCCCTAACCCTCTCCTCGGTCTCGATTCTACGATGTTGCAGCACCATCAGCA	cloning pUAST-attB-V5-Tin
r-Tin	TTGTTGTCTAGAACTACATGTGCTGCATCTGTTGC	cloning pUAST-attB-V5-Tin
f-His-V5-Tin	AACAACCCATGGGCCATCATCACCATCACCACGGTAAGCCTATCCCTAACCC	cloning pET-His-Tin
r2-Tin	AACAACGGTACCCTACATGTGCTGCATCTGTT	cloning pET-His-Tin
f3-Ubx	AACAACGAATTCCCGGAATGAACTCGTACTTTGAAC	cloning pGEX(GST)-Ubx with r-ubx
f-dpp	GCAGCAATTAACCCTCACTAAAGGGGAGTGAATGCGATGGGAAAT	cloning dpp in pGEMTeasy
r-dpp	GCAGCATAATACGACTCACTATAGGGTCCAACATGCTGAGCTTACG	cloning dpp in pGEMTeasy
f-Ubx-Tin BS	CCGCCGCCTACCGACCGTTAATTATCAAGTGGACCGTTAATTATCAAGTGGACCGTTATTATCAAGTGGACCGAGATCTCCGCCG	cloning 3x[Ubx-Tin] binding sites in pGL3-Luciferase

r-Ubx-Tin BS	CGGCGGAGATCTCGGTCCACTTGATAATTAACGG TCCACTTGATAATTAACGGTCCACTTGATAATTAA CGGTCGGTACCCGGCGG	cloning 3x[Ubx-Tin] binding sites in pGL3-Luciferase
f-HX	AACAACGAATTCCCGGAAATCACACATTCTACCC CTGGAT	cloning of pGEX-Ubx fragments
r-HX+HD	AAATCTAGAGTTACTGGATCTCCTTCTTCAGCTTC	cloning of pGEX-Ubx fragments
r-N-ter	AACTCTAGAGTTAGCTGGCCTGGTGTAAACTGC	cloning of pGEX-Ubx fragments
f-HD	AACAACGAATTCCCGGAACAAATGGTCTGCGAAG ACGCG	cloning of pGEX-Ubx fragments
r-TinN51A	AGCGCCGAGCCTGGAACCAAATCTTC	cloning Tin-N51A
f-TinN51A	GGTTCCAGGCTCGGCGCTACAAATC	cloning Tin-N51A

Supplementary Table 3c: List of oligonucleotides. The letter r states for reverse, f for forward.

SUPPLEMENTARY REFERENCES

1. Chan, S. K., Jaffe, L., Capovilla, M., Botas, J. & Mann, R. S. The DNA binding specificity of Ultrabithorax is modulated by cooperative interactions with extradenticle, another homeoprotein. *Cell* **78**, 603–615 (1994).
2. Boube, M. *et al.* Drosophila melanogaster Hox Transcription Factors Access the RNA Polymerase II Machinery through Direct Homeodomain Binding to a Conserved Motif of Mediator Subunit Med19. *PLoS genetics* **10**, e1004303 (2014).
3. Domsch, K. *et al.* The Hox transcription factor Ubx stabilizes lineage commitment by suppressing cellular plasticity in Drosophila. *eLife* **8**, (2019).
4. Steiner, F. A., Talbert, P. B., Kasinathan, S., Deal, R. B. & Henikoff, S. Cell-type-specific nuclei purification from whole animals for genome-wide expression and chromatin profiling. *Genome Research* **22**, 766–777 (2012).
5. Hessinger, C., Technau, G. M. & Rogulja-Ortmann, A. The *Drosophila* Hox gene *Ultrabithorax* acts in both muscles and motoneurons to orchestrate formation of specific neuromuscular connections. *Development* **144**, 139–150 (2017).
6. Monedero Cobeta, I., Salmani, B. Y. & Thor, S. Anterior-Posterior Gradient in Neural Stem and Daughter Cell Proliferation Governed by Spatial and Temporal Hox Control. *Curr. Biol.* **27**, 1161–1172 (2017).
7. Karlsson, D., Baumgardt, M. & Thor, S. Segment-specific neuronal subtype specification by the integration of anteroposterior and temporal cues. *PLoS Biol.* **8**, e1000368 (2010).
8. Cenci, C. & Gould, A. P. Drosophila Grainyhead specifies late programmes of neural proliferation by regulating the mitotic activity and Hox-dependent apoptosis of neuroblasts. *Development* **132**, 3835–3845 (2005).
9. Jin, H. *et al.* Genome-Wide Screens for In Vivo Tinman Binding Sites Identify Cardiac Enhancers with Diverse Functional Architectures. *PLoS Genetics* **9**, e1003195 (2013).
10. Nevil, M., Bondra, E. R., Schulz, K. N., Kaplan, T. & Harrison, M. M. Stable Binding of the Conserved Transcription Factor Grainy Head to its Target Genes Throughout *Drosophila melanogaster* Development. *Genetics* **205**, 605–620 (2017).

11. Roux, K. J., Kim, D. I., Raida, M. & Burke, B. A promiscuous biotin ligase fusion protein identifies proximal and interacting proteins in mammalian cells. *The Journal of Cell Biology* **196**, 801–810 (2012).

12. Kim, D. I. *et al.* An improved smaller biotin ligase for BioID proximity labeling. *Molecular Biology of the Cell* **27**, 1188–1196 (2016).

13. Baker, R. & Schubiger, G. Autonomous and nonautonomous Notch functions for embryonic muscle and epidermis development in Drosophila. *Development* **122**, 617–626 (1996).

14. Yao, K. M. & White, K. Neural specificity of elav expression: defining a Drosophila promoter for directing expression to the nervous system. *J. Neurochem.* **63**, 41–51 (1994).

15. Sanson, B., White, P. & Vincent, J.-P. Uncoupling cadherin-based adhesion from wingless signalling in Drosophila. *Nature* **383**, 627–630 (1996).

16. Venken, K. J. T. *et al.* MiMIC: a highly versatile transposon insertion resource for engineering Drosophila melanogaster genes. *Nature Methods* **8**, 737–743 (2011).

17. Grell, R. F. Non Random Assortment of Non-Homologous Chromosomes in Drosophila Melanogaster. *Genetics* **44**, 421–435 (1959).

18. Azpiazu, N. & Frasch, M. tinman and bagpipe: two homeo box genes that determine cell fates in the dorsal mesoderm of Drosophila. *Genes Dev.* **7**, 1325–1340 (1993).

19. Kennison, J. A. & Tamkun, J. W. Dosage-dependent modifiers of polycomb and antennapedia mutations in Drosophila. *Proceedings of the National Academy of Sciences* **85**, 8136–8140 (1988).

20. Huet, F. *et al.* A deletion-generator compound element allows deletion saturation analysis for genomewide phenotypic annotation. *Proceedings of the National Academy of Sciences* **99**, 9948–9953 (2002).

21. Spradling, A. C. *et al.* The Berkeley Drosophila Genome Project gene disruption project: Single P-element insertions mutating 25% of vital Drosophila genes. *Genetics* **153**, 135–177 (1999).

22. Ostrowski, S., Dierick, H. A. & Bejsovec, A. Genetic control of cuticle formation during embryonic development of Drosophila melanogaster. *Genetics* **161**, 171–182 (2002).

23. Mannervik, M. Control of Drosophila embryo patterning by transcriptional co-regulators. *Experimental Cell Research* **321**, 47–57 (2014).

24. Ray, P. *et al.* Combgap contributes to recruitment of Polycomb group proteins in *Drosophila*. *Proceedings of the National Academy of Sciences* **113**, 3826–3831 (2016).

25. Foo, S. M. *et al.* Zelda Potentiates Morphogen Activity by Increasing Chromatin Accessibility. *Current Biology* **24**, 1341–1346 (2014).

26. Sun, Y. *et al.* Zelda overcomes the high intrinsic nucleosome barrier at enhancers during *Drosophila* zygotic genome activation. *Genome Research* **25**, 1703–1714 (2015).

27. Shi, J. *et al.* Drosophila Brahma complex remodels nucleosome organizations in multiple aspects. *Nucleic Acids Research* **42**, 9730–9739 (2014).

28. Bullock, S. L. & Ish-Horowicz, D. Conserved signals and machinery for RNA transport in Drosophila oogenesis and embryogenesis. *Nature* **414**, 611–616 (2001).

29. Liu, Z. Structural Basis for Recognition of the Intron Branch Site RNA by Splicing Factor 1. *Science* **294**, 1098–1102 (2001).

30. Kennedy, C. F., Krämer, A. & Berget, S. M. A role for SRp54 during intron bridging of small introns with pyrimidine tracts upstream of the branch point. *Mol. Cell. Biol.* **18**, 5425–5434 (1998).
31. Chiu, Y.-F. *et al.* Cwc25 Is a Novel Splicing Factor Required after Prp2 and Yju2 To Facilitate the First Catalytic Reaction. *Molecular and Cellular Biology* **29**, 5671–5678 (2009).
32. Kohtz, J. D. *et al.* Protein-protein interactions and 5'-splice-site recognition in mammalian mRNA precursors. *Nature* **368**, 119–124 (1994).
33. Bauer, R., McGuffin, M. E., Mattox, W. & Tainsky, M. A. Cloning and characterization of the Drosophila homologue of the AP-2 transcription factor. *Oncogene* **17**, 1911–1922 (1998).
34. Coelho, C. M. A. *et al.* A genetic screen for dominant modifiers of a small-wing phenotype in Drosophila melanogaster identifies proteins involved in splicing and translation. *Genetics* **171**, 597–614 (2005).
35. Blencowe, B. J. *et al.* The SRm160/300 splicing coactivator subunits. *RNA* **6**, 111–120 (2000).
36. Negeri, D., Eggert, H., Gienapp, R. & Saumweber, H. Inducible RNA interference uncovers the Drosophila protein Bx42 as an essential nuclear cofactor involved in Notch signal transduction. *Mech. Dev.* **117**, 151–162 (2002).
37. Dobi, K. C., Halfon, M. S. & Baylies, M. K. Whole-Genome Analysis of Muscle Founder Cells Implicates the Chromatin Regulator Sin3A in Muscle Identity. *Cell Reports* **8**, 858–870 (2014).
38. Park, J. W., Parisky, K., Celotto, A. M., Reenan, R. A. & Graveley, B. R. Identification of alternative splicing regulators by RNA interference in Drosophila. *Proc. Natl. Acad. Sci. U.S.A.* **101**, 15974–15979 (2004).
39. Mount, S. M. & Salz, H. K. Pre-messenger RNA processing factors in the Drosophila genome. *J. Cell Biol.* **150**, F37-44 (2000).
40. Müller, J., Gaunt, S. & Lawrence, P. A. Function of the Polycomb protein is conserved in mice and flies. *Development* **121**, 2847–2852 (1995).
41. Zouaz, A. *et al.* The Hox proteins Ubx and AbdA collaborate with the transcription pausing factor M1BP to regulate gene transcription. *The EMBO Journal* **36**, 2887–2906 (2017).
