# Supplementary material for: Multi-level and lineage-specific interactomes of the Hox transcription factor Ubx contribute to its functional specificity
Source: Nat Commun. 2020 Mar 13;11:1388. doi: 10.1038/s41467-020-15223-x (PMC7069958; doi:10.1038/s41467-020-15223-x)
Supplement: Supplementary file 3 — Description of Additional Supplementary Files [file 41467_2020_15223_MOESM3_ESM.docx]

**Description of Supplementary Files**

**File Name: Supplementary Data 1**

**Description:** Unique peptide and LFQ values after filtering of twi-BioID replicates. Unique peptides and LFQ values of the twist-BioID replicates after filtering (see methods).

**File Name: Supplementary Data 2**

**Description:** Unique peptide and LFQ values after filtering of elav-BioID replicates. Unique peptides and LFQ values of the elav-BioID replicates after filtering (see methods).

**File Name: Supplementary Data 3**

**Description:** Unique peptide and LFQ values after filtering of sca-BioID replicates. Unique peptides and LFQ values of the scabrous-BioID replicates after filtering (see methods).

**File Name: Supplementary Data 4**

**Description:** Protein enrichment based on LFQ ratio for WT-replicate 1 of twi-BioID. Protein enrichment based on log2 LFQ ratio of WT over GFP for the replicate 1 of twist-BioID samples. The summary is presented in Supplementary Table 1a.

**File Name: Supplementary Data 5**

**Description:** Protein enrichment based on LFQ ratio for WT-replicate 2 of twi-BioID. Protein enrichment based on log2 LFQ ratio of WT over GFP for the replicate 2 of twist-BioID samples. The summary is presented in Supplementary Table 1a.

**File Name: Supplementary Data 6**

**Description:** Protein enrichment based on LFQ ratio for WT-replicate 3 of twi-BioID. Protein enrichment based on log2 LFQ ratio of WT over GFP for the replicate 3 of twist-BioID samples. The summary is presented in Supplementary Table 1a.

**File Name: Supplementary Data 7**

**Description:** Protein enrichment based on LFQ ratio for WT-replicate 4 of twi-BioID. Protein enrichment based on log2 LFQ ratio of WT over GFP for the replicate 4 of twist-BioID samples. The summary is presented in Supplementary Table 1a.

**File Name: Supplementary Data 8**

**Description:** Protein enrichment based on LFQ ratio for N51A-replicate 1 of twi-BioID. Protein enrichment based on log2 LFQ ratio of N51A over GFP for the replicate 1 of twist-BioID samples. The summary is presented in Supplementary Table 1a.

**File Name: Supplementary Data 9**

**Description:** Protein enrichment based on LFQ ratio for N51A-replicate 2 of twi-BioID. Protein enrichment based on log2 LFQ ratio of N51A over GFP for the replicate 2 of twist-BioID samples. The summary is presented in Supplementary Table 1a.

**File Name: Supplementary Data 10**

**Description:** Protein enrichment based on LFQ ratio for N51A-replicate 3 of twi-BioID. Protein enrichment based on log2 LFQ ratio of N51A over GFP for the replicate 3 of twist-BioID samples. The summary is presented in Supplementary Table 1a.

**File Name: Supplementary Data 11**

**Description:** Protein enrichment based on LFQ ratio for N51A-replicate 4 of twi-BioID. Protein enrichment based on log2 LFQ ratio of N51A over GFP for the replicate 4 of twist-BioID samples. The summary is presented in Supplementary Table 1a.

**File Name: Supplementary Data 12**

**Description:** Protein enrichment based on LFQ ratio for WT-replicate 1 of elav-BioID. Protein enrichment based on log2 LFQ ratio of WT over GFP for the replicate 1 of elav-BioID samples. The summary is presented in Supplementary Table 1b.

**File Name: Supplementary Data 13**

**Description:** Protein enrichment based on LFQ ratio for WT-replicate 2 of elav-BioID. Protein enrichment based on log2 LFQ ratio of WT over GFP for the replicate 2 of elav-BioID samples. The summary is presented in Supplementary Table 1b.

**File Name: Supplementary Data 14**

**Description:** Protein enrichment based on LFQ ratio for WT-replicate 3 of elav-BioID. Protein enrichment based on log2 LFQ ratio of WT over GFP for the replicate 3 of elav-BioID samples. The summary is presented in Supplementary Table 1b.

**File Name: Supplementary Data 15**

**Description:** Protein enrichment based on LFQ ratio for WT-replicate 4 of elav-BioID. Protein enrichment based on log2 LFQ ratio of WT over GFP for the replicate 4 of elav-BioID samples. The summary is presented in Supplementary Table 1b.

**File Name: Supplementary Data 16**

**Description:** Protein enrichment based on LFQ ratio for N51A-replicate 1 of elav-BioID. Protein enrichment based on log2 LFQ ratio of N51A over GFP for the replicate 1 of elav-BioID samples. The summary is presented in Supplementary Table 1b.

**File Name: Supplementary Data 17**

**Description:** Protein enrichment based on LFQ ratio for N51A-replicate 2 of elav-BioID. Protein enrichment based on log2 LFQ ratio of N51A over GFP for the replicate 2 of elav-BioID samples. The summary is presented in Supplementary Table 1b.

**File Name: Supplementary Data 18**

**Description:** Protein enrichment based on LFQ ratio for N51A-replicate 3 of elav-BioID. Protein enrichment based on log2 LFQ ratio of N51A over GFP for the replicate 3 of elav-BioID samples. The summary is presented in Supplementary Table 1b.

**File Name: Supplementary Data 19**

**Description:** Protein enrichment based on LFQ ratio for N51A-replicate 4 of elav-BioID. Protein enrichment based on log2 LFQ ratio of N51A over GFP for the replicate 4 of elav-BioID samples. The summary is presented in Supplementary Table 1b.

**File Name: Supplementary Data 20**

**Description:** Protein enrichment based on LFQ ratio for WT-replicate 1 of sca-BioID. Protein enrichment based on log2 LFQ ratio of WT over GFP for the replicate 1 of scabrous-BioID samples. The summary is presented in Supplementary Table 1c.

**File Name: Supplementary Data 21**

**Description:** Protein enrichment based on LFQ ratio for WT-replicate 2 of sca-BioID. Protein enrichment based on log2 LFQ ratio of WT over GFP for the replicate 2 of scabrous-BioID samples. The summary is presented in Supplementary Table 1c.

**File Name: Supplementary Data 22**

**Description:** Protein enrichment based on LFQ ratio for WT-replicate 3 of sca-BioID. Protein enrichment based on log2 LFQ ratio of WT over GFP for the replicate 3 of scabrous-BioID samples. The summary is presented in Supplementary Table 1c.

**File Name: Supplementary Data 23**

**Description:** Protein enrichment based on LFQ ratio for WT-replicate 4 of sca-BioID. Protein enrichment based on log2 LFQ ratio of WT over GFP for the replicate 4 of scabrous-BioID samples. The summary is presented in Supplementary Table 1c.

**File Name: Supplementary Data 24**

**Description:** Protein enrichment based on LFQ ratio for N51A-replicate 1 of sca-BioID. Protein enrichment based on log2 LFQ ratio of N51A over GFP for the replicate 1 of scabrous-BioID samples. The summary is presented in Supplementary Table 1c.

**File Name: Supplementary Data 25**

**Description:** Protein enrichment based on LFQ ratio for N51A-replicate 2 of sca-BioID. Protein enrichment based on log2 LFQ ratio of N51A over GFP for the replicate 2 of scabrous-BioID samples. The summary is presented in Supplementary Table 1c.

**File Name: Supplementary Data 26**

**Description:** Protein enrichment based on LFQ ratio for N51A-replicate 3 of sca-BioID. Protein enrichment based on log2 LFQ ratio of N51A over GFP for the replicate 3 of scabrous-BioID samples. The summary is presented in Supplementary Table 1c.

**File Name: Supplementary Data 27**

**Description:** Protein enrichment based on LFQ ratio for N51A-replicate 4 of sca-BioID. Protein enrichment based on log2 LFQ ratio of N51A over GFP for the replicate 4 of scabrous-BioID samples. The summary is presented in Supplementary Table 1c.

**File Name: Supplementary Data 28**

**Description:** List, comparison and ranking of proteins enriched in all WT replicates in twiBioID.

**File Name: Supplementary Data 29**

**Description:** List, comparison and ranking of proteins enriched in all N51A replicates in twi-BioID.

**File Name: Supplementary Data 30**

**Description:** List, comparison and ranking of proteins enriched in all WT replicates in elavBioID.

**File Name: Supplementary Data 31**

**Description:** List, comparison and ranking of proteins enriched in all N51A replicates in elav-BioID.

**File Name: Supplementary Data 32**

**Description:** List, comparison and ranking of proteins enriched in all WT replicates in scaBioID.

**File Name: Supplementary Data 33**

**Description:** List, comparison and ranking of proteins enriched in all N51A replicates in sca-BioID.

**File Name: Supplementary Data 34**

**Description:** Comparison of BioID from all tissue using all replicates.

**File Name: Supplementary Data 35**

**Description:** Comparison of BioID data and tissue-specific transcriptome.

**File Name: Supplementary Data 36**

**Description:** List of proteins enriched per nuclear compartment and annotated for mesoderm.

**File Name: Supplementary Data 37**

**Description:** List of proteins enriched per nuclear compartment and annotated for neural system.

**File Name: Supplementary Data 38**

**Description:** List of proteins enriched per nuclear compartment and annotated for neuroectoderm.

**File Name: Supplementary Data 39**

**Description:** Summary of interaction studies of selected partners and associated references. The table contains the list of the partners selected and validated by coIP in cells, embryos, immunostainings from embryos as well as the quantification summary of the coIP performed in cells with GFP-Ubx WT or N51A. The raw data are available in the source file and the table referred to figure 3 and supplementary figure 5 and 6.
