# Supplementary material for: Multi-level and lineage-specific interactomes of the Hox transcription factor Ubx contribute to its functional specificity
Source: Nat Commun. 2020 Mar 13;11:1388. doi: 10.1038/s41467-020-15223-x (PMC7069958; doi:10.1038/s41467-020-15223-x)
Supplement: Supplementary file 43 — Reporting Summary [file 41467_2020_15223_MOESM43_ESM.pdf]

nature research

Corresponding author(s): Ingrid Lohmann

Last updated by author(s): Feb 17, 2020

Reporting Summary

Nature Research wishes to improve the reproducibility of the work that we publish. This form provides structure for consistency and transparency in reporting. For further information on Nature Research policies, see Authors & Referees and the Editorial Policy Checklist .

Statistics

For all statistical analyses, confirm that the following items are present in the figure legend, table legend, main text, or Methods section.

n/a	Confirmed	
☐	☒	The exact sample size (*n*) for each experimental group/condition, given as a discrete number and unit of measurement
☐	☒	A statement on whether measurements were taken from distinct samples or whether the same sample was measured repeatedly
☐	☒	The statistical test(s) used AND whether they are one- or two-sided<br>*Only common tests should be described solely by name; describe more complex techniques in the Methods section.*
☐	☒	A description of all covariates tested
☐	☒	A description of any assumptions or corrections, such as tests of normality and adjustment for multiple comparisons
☐	☒	A full description of the statistical parameters including central tendency (e.g. means) or other basic estimates (e.g. regression coefficient) AND variation (e.g. standard deviation) or associated estimates of uncertainty (e.g. confidence intervals)
☐	☒	For null hypothesis testing, the test statistic (e.g. *F*, *t*, *r*) with confidence intervals, effect sizes, degrees of freedom and *P* value noted<br>*Give P values as exact values whenever suitable.*
☒	☐	For Bayesian analysis, information on the choice of priors and Markov chain Monte Carlo settings
☒	☐	For hierarchical and complex designs, identification of the appropriate level for tests and full reporting of outcomes
☐	☒	Estimates of effect sizes (e.g. Cohen's *d*, Pearson's *r*), indicating how they were calculated

Our web collection on statistics for biologists contains articles on many of the points above.

Software and code

Policy information about availability of computer code

Data collection	Proteomic data were collected using Tri-Hybrid Orbitrap Fusion mass spectrometer (Thermo Fisher Scientific). Immunoblotting and EMSA were collected using INTAS imager. Imaging data were collected using LEICA software
Data analysis	Cytoscape3.4.0, Maxquant1.5.5.1, Perseus1.5.5.3, Fiji, Panther webtool, String Webtool, MEMEsuite webtool, PhotoshopCC, BEDtools, illustrator, Prism8/GraphPad. Full description in methods available.

For manuscripts utilizing custom algorithms or software that are central to the research but not yet described in published literature, software must be made available to editors/reviewers. We strongly encourage code deposition in a community repository (e.g. GitHub). See the Nature Research guidelines for submitting code & software for further information.

Data

Policy information about availability of data

All manuscripts must include a data availability statement. This statement should provide the following information, where applicable:

- Accession codes, unique identifiers, or web links for publicly available datasets
- A list of figures that have associated raw data
- A description of any restrictions on data availability

Raw data of MS analysis, Uniprot and contaminant databases and Maxquant files that support the findings of this study have been deposited in PRIDE (https://www.ebi.ac.uk/pride/archive <https://www.ebi.ac.uk/pride/archive>) with the accession code PXD0144818[http://proteomecentral.proteomexchange.org/cgi/GetDataset?ID=PXD014481 <http://proteomecentral.proteomexchange.org/cgi/GetDataset?ID=PXD014481>].
Freely accessible datasets used in the study are listed below:
-ChIP on ChIP of Tin: GSE41628[https://www.ncbi.nlm.nih.gov/geo/query/acc.cgi?acc=GSE41628 <https://www.ncbi.nlm.nih.gov/geo/query/acc.cgi?acc=GSE41628>]
-ChIPseq of Grh: GSE83305[https://www.ncbi.nlm.nih.gov/geo/query/acc.cgi?acc=GSE83305 <https://www.ncbi.nlm.nih.gov/geo/query/acc.cgi?acc=GSE83305>] using 5-6h ChIP-seq collection.

-Tissue-specific transcriptome and upon Ubx depletion: GSE121670[https://www.ncbi.nlm.nih.gov/geo/query/acc.cgi?acc=GSE121670 <https://www.ncbi.nlm.nih.gov/geo/query/acc.cgi?acc=GSE121670>].
-Tissue-specific ChIP-seq of Ubx: GSE121752[https://www.ncbi.nlm.nih.gov/geo/query/acc.cgi?acc=GSE121752 <https://www.ncbi.nlm.nih.gov/geo/query/acc.cgi?acc=GSE121752>].
The source data underlying figs 1, 2, 3, 4, 5 and Supplementary figs 1, 5, 6, 7, 9 are provided as Source Data file. Other raw files are available from the corresponding author upon reasonable request.

Field-specific reporting

Please select the one below that is the best fit for your research. If you are not sure, read the appropriate sections before making your selection.

[x] Life sciences [ ] Behavioural & social sciences [ ] Ecological, evolutionary & environmental sciences

For a reference copy of the document with all sections, see nature.com/documents/nr-reporting-summary-flat.pdf

Life sciences study design

All studies must disclose on these points even when the disclosure is negative.

Sample size	To reach the adequate requirements combining the proteomic analysis, experimental design, and feasibility of the experiment in term of samples collections, four biological replicates were chosen for proteomic studies. It takes into account the biological variability of the system and the ability to determine the significance of protein enrichment in 2 replicates over 4 estimated after further analysis. For the quantity of nuclear extract used for affinity purification, we determined the minimum requirements by analysing preliminary samples processing. For each tissue, the protein quantity necessary for mass spectrometry purification has been adjusted by experimental trials. All in all, it met the criteria of experimental feasibility, statistical enrichment and biological significance.
Data exclusions	Exclusion if any was done for luciferase assay if experimental replicates presented a highly differential value due to defect in reagent injection by the luminometer (3 experimental replicates). No exclusion of the biological replicates was done (3 independent biological replicates, including each 3 experimental replicates).
Replication	For proteomic experiments, replication was developed in "sample size" and further developed in the method subpart. For luciferase assay, standard replication was applied, meaning, 3 experimental replicates performed for 3 independent biological experiments. For immunostaining, several collection of embryos were mixed, inserting natural biological variation and replication was described in detail for each experiment in the method, figure legend as well as source data file. For western blot a standard of 3 independent experiments were applied for cell system and 2 for pulled collection of embryos as living system.
Randomization	Randomization was naturally inserted by the use of pools of embryos collection from different time point, different Drosophila and cells generation or crossing. Samples were generated in a highly randomised fashion as they were always processed separately and at different time point.
Blinding	Blinding was applied for identification of phenotype to genotype of double heterozygous mutant. The embryos and phenotype were merely observed in Cy3 channel related to muscle pattern and counted without any selection.

Reporting for specific materials, systems and methods

We require information from authors about some types of materials, experimental systems and methods used in many studies. Here, indicate whether each material, system or method listed is relevant to your study. If you are not sure if a list item applies to your research, read the appropriate section before selecting a response.

Materials & experimental systems

n/a	Involved in the study	
[ ]	[x]	Antibodies
[ ]	[x]	Eukaryotic cell lines
[x]	[ ]	Palaeontology
[ ]	[x]	Animals and other organisms
[x]	[ ]	Human research participants
[x]	[ ]	Clinical data

Methods

n/a	Involved in the study	
[x]	[ ]	ChIP-seq
[x]	[ ]	Flow cytometry
[x]	[ ]	MRI-based neuroimaging

Antibodies

Antibodies used	The antibodies used in this study were:<br>-Histone 3, Abcam, reference: 1791,<br>-GFP, Life Technologies, reference: A11122,<br>-Streptavidin-HRP, GE-healthcare, reference: RPN1231,

-Tubulin, Serotec/Biorad, reference: MCA77G,
-HA, Cell Signaling, reference: 3724,
-GST, Cell Signaling, reference: 2624,
-Flag-M2, Sigma, reference: F1804,
-V5, Cell Signaling, reference:13202,
-MBP, Cell Signaling, reference: 2396,
-Myc, Santa Cruz, reference: SC40,
-Beta-Galactosidase, Promega, reference Z3783,
-Tropomyosin 1 (Tm1), Abcam, reference: ab50567,
-Ubx, home-made, Ingrid Lohmann lab (COS-Heidelberg University)
-Deadpan (Dpn), originally from Jürgen Knoblich (IMBA, Austria), generously provided by Ana Rogulja-Ortmann (COS, Heidelberg).
-Grainy Head (Grh), generously provided by Bill McGinnis (UCSD, San Diego),
-Tinman (Tin), generously provided by Manfred Frasch (FAU Department Biology, Erlangen),
-Zelda (Zld), generously provided by Julia Zeitlinger, (Stowers Institute for Medical Research, Missouri),
-C-terminal Binding Protein (CtBP), generously provided by David Arnosti (Michigan State University),
-Combgap (Cg), generously provided by William Brook (University of Calgary, Canada),
-Med19, generously provided by Muriel Boube (Paul Sabatier University - Toulouse III),
-Polycomb (Pc), generously provided by Jürg Müller (MPI of Biochemistry, Munich),
-Motif Binding Protein (M1BP), originally from D. Gilmour (Center for Eukaryotic Gene Regulation, Pennsylvania) generously provided by Andy Saurin (IBDM, Marseille),
-Engrailed 4D9 anti-engrailed/invected was deposited to the DSHB by Goodman, C. (DSHB Hybridoma Product 4D9 anti-engrailed/invected),
-1D4 anti-Fasciclin 2 was deposited to the DSHB by Goodman, C. (DSHB Hybridoma Product 1D4 anti-Fasciclin 2),
-Rat-Elav-7E8A10 anti-elav was deposited to the DSHB by Rubin, G.M. (DSHB Hybridoma Product Rat-Elav-7E8A10 anti-elav),

Validation

Validation acronyms: Immunoprecipitation (IP), immunofluorescence (IF), Western-blot (WB), ImmunoHistoChemistry (IHC).
-Histone 3, statement from Abcam: validated for IHC-Fr, CHIPseq, Dot blot, flow cytometry, IHC-P, Electron Microscopy, ICC/IF, ChIP, IP, WB, ChIP/Chip, IHC-Wholemount, ICC
-GFP, statement from Life Technologies: validated for IF, WB.
-Streptavidin-HRP, statement from GE-healthcare: validated for ELISA, WB.
-Tubulin, statement from Serotec-Biorad: validated for ELISA, IF, IHC, IP, Radio-immunoassay and WB.
-HA, statement from Cell Signaling: validated for WB, IP, IHC, IF, flow cytometry, ChIP.
-GST, statement from Cell Signaling: validated for WB, IP, IF.
-Flag-M2, statement from Sigma: validated for WB, IP, IF.
-V5, statement from Cell Signaling: validated for WB, IP, IF, flow cytometry.
-MBP, statement from Cell Signaling: validated for WB, IP, IF.
-Myc, statement from Santa Cruz: validated for WB, IP, IF, IHC, flow cytometry and ELISA.
-Tm1, statement from Abcam: validated for WB, IHC-Fr, Electron Microscopy, ICC/IF, IHC – Wholemount.
-Beta-Galactosidase, statement from Promega: validated for Immunoprecipitation (IP), WB, Dot blot, IF.
-Ubx produced and validated by ChIP, IF in Domsch K, Carnesecchi J, Disela V, et al. The Hox transcription factor Ubx stabilizes lineage commitment by suppressing cellular plasticity in Drosophila. Elife. 2019;8:e42675. Published 2019 May 3. doi:10.7554/eLife.42675.
-Dpn validated by IF in Eroglu E, Burkard TR, Jiang Y, Saini N, Homem CCF, Reichert H, Knoblich JA (2014) SWI/SNF complex prevents lineage reversion and induces temporal patterning in neural stem cells. Cell 156: 1259–1273. DOI:10.1016/j.cell.2014.01.053.
-Grh, validated by IF in Kim M, McGinnis W. Phosphorylation of Grainy head by ERK is essential for wound-dependent regeneration but not for development of an epidermal barrier. Proc Natl Acad Sci U S A. 2011;108(2):650–655. doi:10.1073/pnas.1016386108.
-Tin, produced and validated by ChIP, IF in Yin Z, Xu X-L, Frasch M (1997) Regulation of the Twist target gene tinman by modular cis-regulatory elements during early mesoderm development. Development 124: 4871–4982.
-Zelda validated by ChIP, IF in Nien CY, Liang HL, Butcher S, et al. Temporal coordination of gene networks by Zelda in the early Drosophila embryo. PLoS Genet. 2011;7(10):e1002339. doi:10.1371/journal.pgen.1002339.
-CtBP produced and validated by IF in Mani-Telang P, Arnosti DN. Developmental expression and phylogenetic conservation of alternatively spliced forms of the C-terminal binding protein corepressor. Dev Genes Evol. 2007;217(2):127–135. doi:10.1007/s00427-006-0121-4.
-Cg produced and validated by IF, IHC WB, ChIP in Svendsen PC, Marshall SD, Kyba M, Brook WJ. The combgap locus encodes a zinc-finger protein that regulates cubitus interruptus during limb development in Drosophila melanogaster. Development. 2000 Oct; 127(19):4083-93., and further on by WB, ChIP in Ray P, De S, Mitra A, et al. Combgap contributes to recruitment of Polycomb group proteins in Drosophila. Proc Natl Acad Sci U S A. 2016;113(14):3826–3831. doi:10.1073/pnas.1520926113.
-Med19 produced and validated by IF in Boube M, Hudry B, Immarigeon C, et al. Drosophila melanogaster Hox transcription factors access the RNA polymerase II machinery through direct homeodomain binding to a conserved motif of mediator subunit Med19. PLoS Genet. 2014;10(5):e1004303. Published 2014 May 1. doi:10.1371/journal.pgen.1004303.
-Pc produced and validated by ChIP in Papp B, Müller J. Histone trimethylation and the maintenance of transcriptional ON and OFF states by trxG and PcG proteins. Genes Dev. 2006;20(15):2041–2054. doi:10.1101/gad.388706.
-M1BP produced in Li J, Gilmour DS. Distinct mechanisms of transcriptional pausing orchestrated by GAGA factor and M1BP, a novel transcription factor. EMBO J. 2013;32(13):1829–1841. doi:10.1038/emboj.2013.111 and validated by ChIP and WB in

Zouaz A, Auradkar A, Delfini MC, et al. The Hox proteins Ubx and AbdA collaborate with the transcription pausing factor M1BP to regulate gene transcription. EMBO J. 2017;36(19):2887–2906. doi:10.15252/embj.20169575.
-Engrailed, initially developed in Expression of engrailed proteins in arthropods, annelids, and chordates. Goodman CS Cell 58.5 (1989 Sep 8): 955-68., validated for immunofluorescence (IF), Western-blot (WB), ImmunoHistoChemistry (IHC), Gel supershift.
-Fasciclin2, initially developed in Drosophila Futsch/22C10 is a MAP1B-like protein required for dendritic and axonal development.Klämbt C Neuron 26.2 (2000 May): 357-70., validated for ELISA, IF, IHC, WB.
-Elav, initially developed in The activities of two Ets-related transcription factors required for Drosophila eye development are modulated by the Ras/MAPK pathway. Rubin GM Cell 78.1 (1994 Jul 15): 137-47., validated for IF, IHC.

Eukaryotic cell lines

Policy information about cell lines

Cell line source(s)	S2R+ Drosophila cell line
Authentication	Generous gift from the Tobias Dick lab (DKFZ), acquired from the Drosophila Genomics Center Resource
Mycoplasma contamination	line tested negative for mycoplasma
Commonly misidentified lines (See ICLAC register)	not applicable

Animals and other organisms

Policy information about studies involving animals; ARRIVE guidelines recommended for reporting animal research

Laboratory animals	Drosophila melanogaster. The lines are listed in the Table 3
Wild animals	Not applicable
Field-collected samples	Fly are maintained at 22°C. For proteomic and BioID related staining, embryos collections were staged at 29°C as described in the method subpart. For other experiment, embryos collection were performed at 22-25°C.
Ethics oversight	Ethical approvance is not applicable for Drosophila melanogaster specie. Transgenic fly line were controlled by the Institutional BioSafety Committee.

Note that full information on the approval of the study protocol must also be provided in the manuscript.
